# Gut Bacteriome Analysis of *Anastrepha fraterculus* sp. 1 During the Early Steps of Laboratory Colonization

**DOI:** 10.3389/fmicb.2020.570960

**Published:** 2020-10-20

**Authors:** Julieta Salgueiro, Lida E. Pimper, Diego F. Segura, Fabián H. Milla, Romina M. Russo, Elias Asimakis, Panagiota Stathopoulou, Kostas Bourtzis, Jorge L. Cladera, George Tsiamis, Silvia B. Lanzavecchia

**Affiliations:** ^1^Laboratorio de Insectos de Importancia Agronómica, Instituto de Genética “E.A. Favret”, Centro de Investigación en Ciencias Veterinarias y Agronómicas – Instituto Nacional de Tecnología Agropecuaria, Instituto de Agrobiotecnología y Biología Molecular – Consejo Nacional de Investigaciones Científicas y Tecnológicas, Buenos Aires, Argentina; ^2^Consejo Nacional de Investigaciones Científicas y Técnicas, Buenos Aires, Argentina; ^3^Department of Environmental Engineering, University of Patras, Agrinio, Greece; ^4^Insect Pest Control Laboratory, Joint FAO/IAEA Programme of Nuclear Techniques in Food and Agriculture, Vienna, Austria

**Keywords:** NGS, taxonomic identification, core bacteriome, bacterial diversity, bacterial richness, fruit fly, SIT

## Abstract

Microbial communities associated to insect species are involved in essential biological functions such as host nutrition, reproduction and survivability. Main factors have been described as modulators of gut bacterial community, such as diet, habit, developmental stage and taxonomy of the host. The present work focuses on the complex changes that gut microbial communities go through when wild insects are introduced to artificial rearing conditions. Specifically, we analyzed the effect of the laboratory colonization on the richness and diversity of the gut bacteriome hosted by the fruit fly pest *Anastrepha fraterculus* sp. 1. Bacterial profiles were studied by amplicon sequencing of the 16S *rRNA* V3–V4 hypervariable region in gut samples of males and females, in teneral (1-day-old, unfed) and post-teneral (15-day-old, fed) flies. A total of 3,147,665 sequence reads were obtained and 32 bacterial operational taxonomic units (OTUs) were identified. Proteobacteria was the most abundant phylum (93.3% of the total reads) and, *Wolbachia* and *Enterobacter* were the most represented taxa at the genus level (29.9% and 27.7%, respectively, of the total read counts). Wild and laboratory flies showed highly significant differences in the relative abundances of bacteria. The analysis of the core bacteriome showed the presence of five OTUs in all samples grouped by origin, while nine and five OTUs were exclusively detected in laboratory and wild flies, respectively. Irrespective of fly origin or sex, a dominant presence of *Wolbachia* was observed in teneral flies, whereas *Enterobacter* was highly abundant in post-teneral individuals. We evidenced significant differences in bacterial richness and diversity among generations under laboratory colonization (F0, F1, F3 and F6) and compared to laboratory and wild flies, displaying also differential patterns between teneral and post-teneral flies. Laboratory and wild *A. fraterculus* sp. 1 harbor different gut bacterial communities. Laboratory colonization has an important effect on the microbiota, most likely associated to the combined effects of insect physiology and environmental conditions (e.g., diet and colony management).

## Introduction

Insects can be considered as multiorganismal entities. Their microbiota accounts for up to 10% of the insect’s biomass and is involved in essential physiological roles modulating host fitness (Zilber-Rosenberg and Rosenberg, 2008; Feldhaar, 2011; Douglas, 2015; Morris, 2018; Carthey et al., 2019; Brown et al., 2020). Insect gut microbiome comprises obligate and facultative symbionts, opportunistic parasites, and mutualistic microbes (Bourtzis and Miller, 2003; Pontes and Dale, 2006), a complex structure that is mainly affected by host environment and taxonomy (Colman et al., 2012; Engel and Moran, 2013; Augustinos et al., 2019). Gut bacteria are particularly involved in metabolic pathways that enable the host to utilize nutrient-poor or unbalanced diets, providing essential amino acids (EAAs), vitamins, lipids and co-factors (Dillon and Dillon, 2004; Wu et al., 2006; Ankrah et al., 2017). Bacteria also participate in enzymatic functions, such as, hydrolysate of xylan, lipids, and esters, or fermentation of complex polysaccharides (Engel and Moran, 2013). In addition, gut bacteria are able to perform degradation of natural toxins such us phenolic compounds and complex terpenoids involved in plant defense (Hammer and Bowers, 2015; Berasategui et al., 2016; Jing et al., 2020) or chemical toxins facilitating host insecticide resistance (Vontas et al., 2011).

Tephritidae (Diptera) is a rather large family (ca. 5000 species, ref.) that includes about 70 species of fruit flies of economic importance. These species are considered pests because larvae develop inside a wide range of fruit species including many commercial ones (White and Elson-Harris, 1992). In most tephritid fruit flies examined so far, Enterobacteriaceae has been recognized as the dominant taxonomic group, also including a range of culturable and unculturable bacteria associated to the different life styles of the host species (Behar et al., 2008; Jurkevitch, 2011; Müller, 2013; Augustinos et al., 2015; Ventura et al., 2018). A greater richness of digestive symbiotic species is associated to polyphagous flies including microbes belonging to Proteobacteria and Firmicutes phyla (Morrow et al., 2015).

Previous studies on tephritids strongly suggested that symbionts associated to the digestive tract positively affect the host fitness. The majority of the studies have focused on the effect of adult and larval diet, host taxonomy and developmental stage (Augustinos et al., 2019; Noman et al., 2020). These studies showed that gut bacteria are able to improve parameters related with survivability and reproduction, such as male sexual performance, flight ability, female fecundity, foraging behavior and longevity under starvation (Ben-Yosef et al., 2008a,b; Ben-Ami et al., 2010; Gavriel et al., 2011; Augustinos et al., 2015; Kyritsis et al., 2017, 2019; Akami et al., 2019; reviewed by Noman et al., 2020). On the other hand, certain gut bacteria have been found to be pathogenic for the host (Behar et al., 2008).

*Anastrepha fraterculus* Wiedemann (Diptera: Tephritidae), known as the South American fruit fly, has a wide geographic distribution ranging from northern Mexico to central Argentina (Steck, 1999). This fruit fly pest is recognized as a complex of cryptic species composed by at least eight morphotypes (Selivon et al., 2005; Hernández-Ortiz et al., 2012, 2015; Devescovi et al., 2014). Particularly in Argentina, only one member of this complex is present, the *A. fraterculus* sp. 1 or Brazilian 1 morphotype (Selivon et al., 2005; Goday et al., 2006; Hernández-Ortiz et al., 2012). This morphotype is considered a serious threat to fruit production and trade in Argentina and several South American countries. Currently, only chemical control strategies or insect traps are implemented to manage the pest in integrated management programs, which prompted the development of specific and environmentally friendly methods such as the sterile insect technique (SIT). The SIT involves the mass rearing, sterilization and release of sterile insects to compete and mate with wild individuals resulting in non-viable offspring and a gradual reduction of the pest population (Metcalf and Luckmann, 1994; Hendrichs and Robinson, 2009). Significant progress has been made to set the basis of knowledge needed to implement the SIT against *A. fraterculus*. In particular, mass rearing protocols (Salles, 1999; Jaldo et al., 2001; Sobrinho et al., 2006; Vera et al., 2007; Hernández et al., 2010; Walder et al., 2014), irradiation procedures (Allinghi et al., 2007a,b) and behavioral and physiological studies to improve the competitiveness of sterile males (Segura et al., 2007, 2009, 2013; Abraham et al., 2011, 2013; Liendo et al., 2013; Vera et al., 2013; Bachmann et al., 2015, 2017, 2019) have been addressed. Nonetheless, previous studies seldom considered *A. fraterculus* as a multiorganismal entity and only Juárez et al. (2019) focused on the importance of gut bacteria in *A. fraterculus* sp. 1 physiology and behavior.

The implementation of a successful SIT strategy requires that insects adapt to mass rearing conditions without losing valuable traits that allow them to survive and mate in natural conditions (Ochieng-Odero, 1994; Cayol, 2000; Meats et al., 2009). During the introduction of a wild population to artificial rearing conditions (laboratory colonization), variations in the environment and diet could affect biological parameters of flies and the presence and abundance of symbionts that are key determinants of their sexual competitiveness and survival (Bartlett, 1984; Cayol, 2000). In natural habitats, the development of immature stages of fruit fly species takes place first inside the fruit and then, after pupation, buried into the ground. In laboratory conditions, the eggs are deposited by females in artificial oviposition units (OUs) then transferred to artificial larval diet and pupation substrate (Walder et al., 2014). Likewise, under laboratory rearing, adult flies are provided artificial food (including a protein supply and water offered *ad libitum*), which extensively differs from the available resources of the wild environment. Such artificial conditions (among others) could affect gut bacterial diversity and the vertical transmission of symbionts from parents to offspring, typically from mothers through the eggs when females are forced to lay eggs in artificial oviposition devices (Lauzon et al., 2009; Jaenike, 2012). Likewise, artificial rearing media (both at larval and adult stages) can potentially affect the diversity of bacteria in the gut, favoring the presence of specific taxonomic groups that are not common in nature.

In the present work, we hypothesized that the composition of gut bacterial taxa hosted by *A. fraterculus* sp. 1 is modified during the laboratory colonization and vary among diverse origins, maintaining a shared central core of bacteria. Our approach included the analysis of bacterial diversity and abundance regarding sex, feeding status and origin, studied in adult insects from a wild population, flies through the first six generations during the laboratory colonization process, and flies reared under laboratory conditions.

## Materials and Methods

### Laboratory Insects

Adult insects were obtained from the *A. fraterculus* colony kept at the Institute of Genetics “Ewald A. Favret” (IGEAF). This colony (hereafter LAB flies) was established in 2007 and maintained for ca. 60 generations. No wild material has been introduced to refresh its genetic background. This fruit fly strain derived from a colony kept at Estación Experimental Agroindustrial Obispo Colombres (Tucumán, Argentina) established in 1997 with wild pupae recovered from infested guavas (*Psidium guajava*, Myrtaceae) collected in Tafi Viejo (Tucumán, Argentina). Flies used in the present study were collected at the pupal stage. After emergence, adult insects were handled in the same way as described above and kept under the same experimental conditions.

### Wild Insects Collected From Traps

Adult individuals of *A. fraterculus* sp. 1 were collected using McPhail traps lured with torula yeast. Traps were hanged from an ubajay tree (*Hexachlamys edulis*, Myrtaceae), located within INTA (Instituto Nacional de Tecnología Agropecuaria) experimental field (34°36′24.7″S 58°40′07.6″W), during the fruiting season (December) and were daily checked. Flies were collected with an aspirator and then taken to the laboratory, sorted by sex and their digestive tract immediately dissected (see below). The samples were named as WU (wild ubajay) (Supplementary Table S1).

### Wild Insects Collected From Fruits

Feijoa (*Acca sellowiana*, syn. *Feijoa sellowiana*, Myrtaceae) fruits infested with *A. fraterculus* sp. 1 larvae were sampled in Hurlingham, Buenos Aires, Argentina (34°36′40.2″S 58°40′20.9″W). Fruits were transported to the laboratory and placed on perforated plastic trays which fit in larger trays with sand as substrate for pupation. Twice a week, pupae were recovered and transferred to 3-liter glass containers and kept under controlled environmental conditions (temperature [temp]: 25 ± 1°C; relative humidity [RH]: 70 ± 10%) until adult emergence.

**FIGURE 1:**
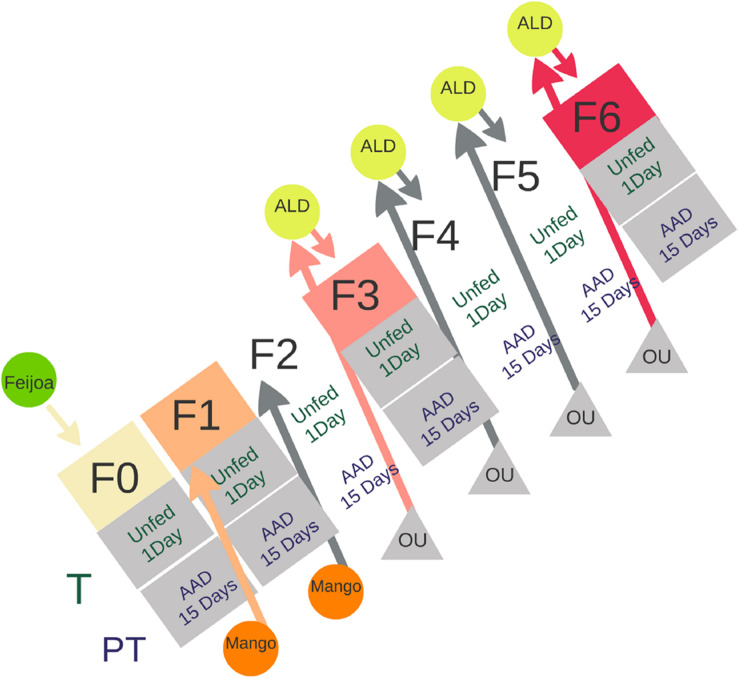
Schematic description of the protocol followed to establish an *A. fraterculus* sp. 1 laboratory colony using wild flies recovered from feijoa infested fruits (green circle). F0 to F6 indicate the generations of flies reared under laboratory conditions. T (teneral) and PT (post-teneral) individuals, represent feeding states of adult insects. Mango and artificial oviposition units (OUs) were used to collect eggs for the next generation. ALD, artificial larval diet; AAD, artificial adult diet.

Emerged adults were identified at the species level according to their morphology following Hernández-Ortiz et al. (2010) and Norrbom et al. (2012). Those flies that belong to *A. fraterculus* were placed in standard cages under controlled conditions (temp: 25°C ± 1°C; RH: 75 ± 5%; photoperiod: 14:10 [light:dark]) with water but no food. Fifteen adult individuals of each sex (males and females) were randomly sampled from the cage in two different feeding status: unfed individuals collected the first day after emergence (from now on called “teneral” [T] flies) and, 15-day-old (sexually mature and fed) individuals (from now on called “post-teneral” [PT] flies) (Figure 1). Post teneral flies were provided with water and artificial adult diet (a mix of hydrolyzed yeast:hydrolyzed corn:sugar in a 1:2:4 ratio, and vitamins [Dayamineral, Abbott]).

Wild flies that emerged in the laboratory were considered the first generation of artificial rearing (F0). This population was used to reproduce insects and obtain the following generations (F1 to F6) (Figure 1). In order to offer an adequate oviposition substrate, females from F0 to F2 generations were provided ripe mango fruits (*Mangifera indica*, Anacardiaceae). Mangoes were washed with neutral detergent and tap water and then immersed in a 0.06% w/v sodium benzoate solution before exposure to females. Exposure lasted 24 h. Fruit were then placed in sealed boxes with a layer of vermiculite as pupation substrate. Pupae were recovered two times per week and used to establish the next generation. Further generations (F3 to F6) were obtained using artificial OUs according to Parreño et al. (2014) (Supplementary Table S1). OUs consisted of cylindrical plastic vials (2 cm in height, 2.5 cm in diameter) filled with water colored with red food dye (Fleibor, Tablada, Buenos Aires, Argentina) and covered with Parafilm M (Pechiney Plastic Packaging, Chicago, IL, United States). After 24–48 h, eggs were recovered from the OUs and transferred to containers (4 cm × 2.5 cm × 3 cm) filled with artificial larval diet (Percent composition [% w/v] of the following supplies: Torula type B (Bioserve Inc.) 6.0; sugar 6.0; wheat germ 6.0; cholesterol 0.05; agar–agar 3.0; methyl paraben 0.1; Be Na 0.1; water 70 to 80; and HCl 36% 0.5 ml to maintain the pH between 3.7 and 4.0 [Salles, 1999]). These containers were placed over a thin layer of vermiculite as pupation substrate. Pupae were recovered two times per week and used to establish the next generation.

### Digestive Tract Dissection and DNA Isolation

Adult flies were washed twice in 70% ethanol and sterilized distilled water. The gut and the subesophageal bulb were identified and dissected with sterile dissecting forceps in PBS 1X under a stereoscopic microscope (Olympus SZ30, 40× zoom) according to Caetano et al. (2006). We performed three replicates for each evaluated condition with five guts per sample (see more details in Figure 1 and Supplementary Table S1). We considered three main factors: gender; origin (F0, LAB and WU) and feeding status (1-day-old and unfed individuals, and 15-day-old and fed flies); except in the case of WU for which feeding status was unknown.

Total DNA extractions of gut samples were performed following Juárez et al. (2019) based on the procedure described by Baruffi et al. (1995) with the following modifications related to sample size: (i) all volumes were reduced to half; (ii) final elution was reduced to 10 μL of TE buffer (Tris base 10 mM; EDTA 1 mM, pH 8.1). Briefly, five guts were ground and incubated (65°C) in a lysis buffer (NaCl 100 mM, Sucrose 200 mM, Tris-HCl pH 9.1 100 mM, EDTA 50 mM, SDS 0.5%) with proteinase K (final concentration of 100 μg/ml) (USB). The incubation was stopped by adding potassium acetate 8M and centrifuged. The recovered supernatant was treated with RNase (1 mg/ml) (USB). DNA was precipitated with 100% ethanol and centrifuged, the pellet was washed with 100% ethanol and 70% ethanol, and finally dried and resuspended in TE buffer.

### Library Preparation and Illumina MiSeq Sequencing

The quantity and quality of the extracted DNAs were measured using NanoDrop 1000 (Thermo Fisher Scientific, Wilmington, United States). A quantity of 50 ng of DNA per sample was used as template to generate amplicons corresponding to the V3–V4 hypervariable region of the bacterial 16S *rRNA* gene. A first round of PCR amplification was performed using KAPA HiFi HotStart PCR Kit (Kapa Biosystems) and MiSeq primers 341F and 805R (Klindworth et al., 2013). Negative controls were included in DNA extractions and PCRs were performed under the same conditions as the rest of the samples but without any genetic material. No amplicons were obtained from these negative controls. PCR products obtained were separated in a 1.2% w/v agarose gel electrophoresis to verify the size. The amplification products were visualized in Bio-Rad’s Gel Doc^TM^ XR + system. Positive PCR fragments were then purified from primers and primer dimers using a 20% PEG, 2.5 M NaCl solution, centrifuged at 14.000 × *g* for 20 min and the precipitate was washed twice with 125 μl of a 70% v/v ethanol solution and centrifuged at 14.000 × *g* for 10 min as previously described (Ntougias et al., 2016). The dried precipitates were suspended in 15 μl of sterile deionized water and the concentration was measured with a Quawell Q5000 micro-volume UV-Vis spectrophotometer, diluted up to 10 ng/μl and used as template in a second round of PCR. In this step, indexed adapters were added to the ends of the 16S rDNA amplicons, as well as the Illumina adaptors. The combinatorial use of index primers resulted in unique samples that were pooled and sequenced on one Illumina MiSeq run. The resulting amplicons were cleaned-up by AMPure XP beads (Agencourt, United Kingdom) and diluted to 2.66 ng/μl. Finally, they were pooled equimolarly and mixed into indexed library following the 16S-metagenomic library preparation guide 15044223-b (Illumina Inc., 2013). Massive amplicon sequencing was performed using an Illumina MiSeq sequencing platform by Macrogen.

### Data Analysis

The pre-processing of reads was carried out using USEARCH v10. Paired Fastq files were assembled by using algorithms implemented in USEARCH v10 using -fastq_mergepairs command with -fastq_maxdiffs, -fastq_pctid, -fastq_minmergelen, and -fastq_maxmergelen options set at default values. All reads were trimmed and filtered by quality using -fastq_filter, with the -fastq_maxee option set at 1.0, and -fastx_uniques commands. Unique sequences were identified, and all samples were clustered at increasing similarities of 97% using UPARSE-OTU algorithm (Edgar, 2013). Using this algorithm, chimera filtering and operational taxonomic units (OTUs) clustering were carried out simultaneously. For the clustering, a minimum abundance (value = 2) was used discarding singletons. For the OTU Table trimming, we defined 0.001 as the minimum frequency for an OTU. The OTU frequency was calculated as follow: (number of count reads for an OTU/total number of count reads)^∗^100. For the OTU Table trimming, we defined 0.001 as the minimum frequency for an OTU. The taxonomy assignment was performed against a reference database (SILVA release 119; Quast et al., 2013). UNCROSS2 algorithm was run to detect and filter crosstalk (Edgar, 2018).

Diversity estimates including observed OTUs and Good’s coverage were calculated using final count data. Richness (Chao1), diversity (Simpson and Shannon), dominance (Berger–Parker) and evenness (Pielou) indices of alpha diversity, which reflect the diversity of individual samples were calculated based on “vegan” R package (Oksanen et al., 2019) and were plotted using the “ggplot2” R Package (Wickham, 2016).

Phylogenetic diversity (Faith index) was estimated using the Picante package in R (Kembel et al., 2010). Alpha diversity indices were compared by pairwise Kruskal–Wallis test in R (R Core Team, 2020). Core bacterial OTUs shared by LAB, WU, F0 and F1–F6 were identified by comparing OTUs from the different origins following Andongma et al. (2019).

Beta diversity was analyzed to evaluate the similarity of bacterial communities from different locations using Generalized UniFrac distance (Chen et al., 2012) and visualized via Non-metric Multidimensional scaling (NMDS) plot using the RHEA pipeline in R (Lagkouvardos et al., 2017). A permutational multivariate analysis of variance using distance matrices was calculated using “adonis” function from “vegan” R package to determine significance differences between the separated groups. Statistically significant differences between samples were identified with permutational multivariate analysis of variance (PERMANOVA; Anderson, 2001), *p*-values of PERMANOVA test indicating the significance of group separations and the dissimilarity scale of the grid, *d* = 0.1 means that the distance between two grid lines represent approximately 10% dissimilarity between the samples. The Bonferroni–Hochberg method was used to correct for multiple PERMANOVA testing. The Non-parametric Wilcoxon rank sum test (Kruskal, 1957) was used to perform pair-wise comparisons of mean relative abundance of bacteria between gut samples.

## Results

### Overall Data Analysis

After an ultimate and strict trimming process, a total of 3,147,665 high quality reads were achieved from a total of 66 *A. fraterculus* sp. 1 gut samples. A set of 32 bacterial OTUs were identified, clustered at 97% sequence similarity (Table 1 and Supplementary Table S1). Members of three bacterial phyla were identified: Patescibacteria, Proteobacteria and Firmicutes. Proteobacteria was the most abundant taxonomic group (93.3% of reads), followed by Firmicutes (6.5%) and Patescibacteria (0.2%). Within Proteobacteria, two taxonomic classes dominated: Alpha- and Gammaproteobacteria. At the genus level, *Wolbachia* and *Enterobacter* were the most abundant taxa (29.9% and 27.7% of the obtained reads, respectively), followed by *Providencia* (8.3%), *Aeromonas* (7.1%), *Citrobacter* (5.9%) and *Burkholderia–Caballeronia–Paraburkholderia* (4.4%) (Table 1). Good’s coverage index was 98%, suggesting that the majority of bacterial phylotypes in the insect digestive tract were included in this study. In addition, three OTUs (15, 124 and 19) were placed in three distinct phylogenetic positions, showing lower than 97% similarity to known described species of the genera *Raoultella* sp., *Klebsiella* sp. and the phylum of *Saccharibacteria*, respectively. For OTU19 taxonomic assignment was not feasible below the phylum level.

**TABLE 1:** Representation and classification of OTUs identified in *A. fraterculus* sp. 1 gut bacteriome. The OTUs highlighted compose the *A. fraterculus* sp. 1 gut bacterial core proposed in the present work.

OTU ID	Read counts	% read counts	Phylum	Class	Order	Family	Genus
OTU 1	942423	29.9	Proteobacteria	Alphaproteobacteria	Rickettsiales	Anaplasmataceae	*Wolbachia*
OTU 2	860684	27.3	Proteobacteria	Gammaproteobacteria	Enterobacteriales	Enterobacteriaceae	*Enterobacter*
OTU 3	141265	4.5	Proteobacteria	Gammaproteobacteria	Aeromonadales	Aeromonadaceae	*Aeromonas*
OTU 4	259733	8.3	Proteobacteria	Gammaproteobacteria	Enterobacteriales	Enterobacteriaceae	*Providencia*
OTU 5	67142	2.1	Firmicutes	Bacilli	Lactobacillales	Enterobacteriaceae	*Enterococcus*
OTU 6	72793	2.3	Proteobacteria	Gammaproteobacteria	Betaproteobacteriales	Burkholderiaceae	*Burkholderia–Caballeronia–Paraburkholderia*
OTU 7	32959	1.1	Firmicutes	Bacilli	Bacillales	Staphylococcaceae	*Staphylococcus*
OTU 8	35181	1.1	Proteobacteria	Alphaproteobacteria	Caulobacterales	Caulobacteraceae	*Caulobacter*
OTU 9	64879	2.1	Proteobacteria	Gammaproteobacteria	Betaproteobacteriales	Burkholderiaceae	*Burkholderia–Caballeronia–Paraburkholderia*
OTU 10	81701	2.6	Proteobacteria	Gammaproteobacteria	Aeromonadales	Aeromonadaceae	*Aeromonas*
OTU 11	41643	1.3	Firmicutes	Bacilli	Lactobacillales	Lactobacillaceae	*Lactobacillus*
OTU 12	27726	0.9	Firmicutes	Bacilli	Lactobacillales	Streptococcaceae	*Lactococcus*
OTU 13	29721	0.9	Proteobacteria	Alphaproteobacteria	Rhizobiales	Xanthobacteraceae	*Bradyrhizobium*
OTU 14	20969	0.7	Proteobacteria	Alphaproteobacteria	Sphingomonadales	Sphingomonadaceae	*Sphingomonas*
OTU 15	65801	2.1	Proteobacteria	Gammaproteobacteria	Enterobacteriales	Enterobacteriaceae	*Raoultella*
OTU 16	10523	0.3	Proteobacteria	Gammaproteobacteria	Enterobacteriales	Enterobacteriaceae	*Serratia*
OTU 17	12157	0.4	Proteobacteria	Alphaproteobacteria	Rhizobiales	Rhizobiaceae	*Mesorhizobium*
OTU 18	5227	0.2	Proteobacteria	Gammaproteobacteria	Pseudomonadales	Moraxellaceae	*Acinetobacter*
OTU 19	6261	0.2	Saccharibacteria	Unknown	Unknown	Unknown	Unknown
OTU 20	19166	0.6	Firmicutes	Bacilli	Bacillales	Staphylococcaceae	*Staphylococcus*
OTU 21	5358	0.2	Proteobacteria	Alphaproteobacteria	Rhizobiales	Rhizobiaceae	*Mesorhizobium*
OTU 22	4207	0.1	Proteobacteria	Alphaproteobacteria	Acetobacterales	Acetobacteraceae	*Commensalibacter*
OTU 24	8207	0.3	Firmicutes	Bacilli	Lactobacillales	Streptococcaceae	*Streptococcus*
OTU 26	8951	0.3	Proteobacteria	Gammaproteobacteria	Betaproteobacteriales	Neisseriaceae	*Neisseria*
OTU 31	4088	0.1	Proteobacteria	Gammaproteobacteria	Pseudomonadales	Moraxellaceae	*Acinetobacter*
OTU 35	6508	0.2	Firmicutes	Bacilli	Lactobacillales	Enterobacteriaceae	*Enterococcus*
OTU 44	3323	0.1	Proteobacteria	Gammaproteobacteria	Betaproteobacteriales	Neisseriaceae	*Neisseria*
OTU 52	5783	0.2	Proteobacteria	Gammaproteobacteria	Enterobacteriales	Enterobacteriaceae	*Enterobacter*
OTU 65	184274	5.9	Proteobacteria	Gammaproteobacteria	Enterobacteriales	Enterobacteriaceae	*Citrobacter*
OTU 124	107352	3.4	Proteobacteria	Gammaproteobacteria	Enterobacteriales	Enterobacteriaceae	*Klebsiella*
OTU 136	4119	0.1	Proteobacteria	Gammaproteobacteria	Alteromonadales	Alteromonadaceae	*Rheinheimera*
OTU 168	7541	0.2	Proteobacteria	Gammaproteobacteria	Enterobacteriales	Enterobacteriaceae	*Enterobacter*

### Core Bacteriome Analysis

**FIGURE 2:**
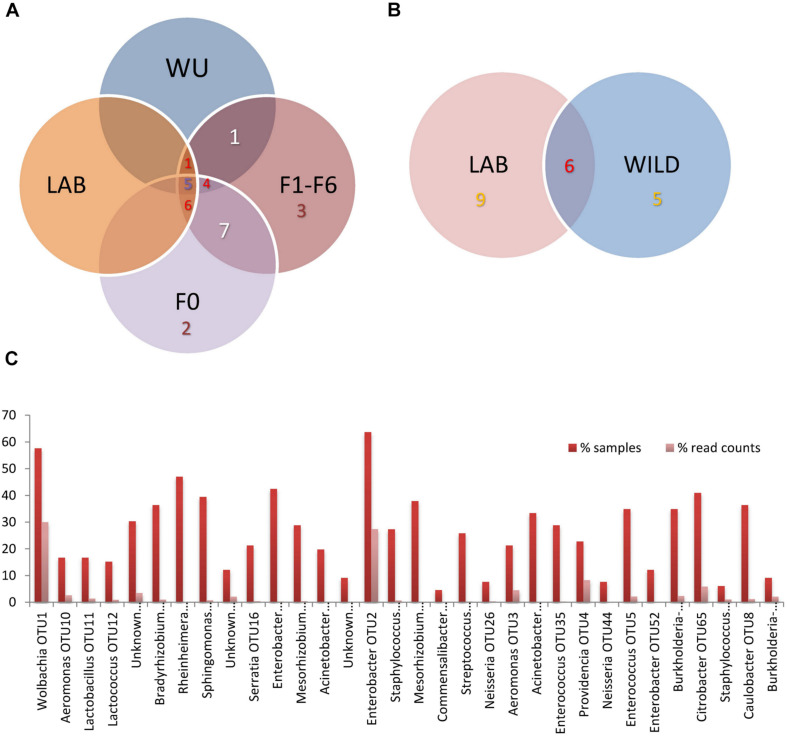
Distribution of bacterial OTUs among *A. fraterculus* sp. 1 gut samples. **(A)** samples pooled in four groups (LAB, WU, F0, and F1–F6) according to their origin. **(B)** Samples grouped in LAB and WILD (F0 + WU). LAB, individuals from the laboratory colony; WU, wild individuals from ubajay with unknown feeding status; F0, individuals collected from infested feijoa fruits; F1–F6, individuals from generations 1, 3 and 6 reared under laboratory conditions. **(C)** OTU representation: % of samples and reads per OTU considering all analyzed samples (*N* = 66) (See more details in Supplementary Tables S2A–D).

A total of five OTUs (*Rheinheimera*, *Enterobacter* [OTU2], *Acinetobacter* [OTU31], *Enterococcus* and *Providencia*) made up the core bacterial community (36.1% of read counts). We defined the core bacteriome of *A. fraterculus* sp. 1 as constituted by taxonomic units present in all the samples grouped by origin (LAB, WU, including samples involved in the laboratory colonization assay F0 and F1–F6) (Figure 2A and Table 1). The relative abundance of taxonomic groups from the core bacteriome varied in the examined samples ranging from 9.5% reads (40% samples) in average for LAB, 7.9% (50% of the samples) in average for WU, 6.1% (50% of the samples in average) in average for F0 and 6.7% (33.3% of the samples) in average for F1–F6 (Figure 2A and Supplementary Tables S2A,B). In addition, when LAB and WU were compared, six OTUs (*Rheinheimera*, *Klebsiella* [OTU124], *Enterobacter* [OTU2], *Acinetobacter* [OTU31], *Enterococcus* and *Providencia*) were shared. Furthermore, nine OTUs (*Wolbachia*, *Bradyrhizobium*, *Sphingomonas*, two OTUs of *Mesorhizobium*, *Acinetobacter* [OTU18], *Staphylococcus*, *Burkholderia–Caballeronia–Paraburkholderia* [OTU6] and *Caulobacter*) were found in LAB but not in WU samples and, five OTUs (*Lactobacillus*, *Lactococcus*, *Raoultella* [OTU15], *Enterobacter* [OTU168] and *Citrobacter*) were detected in WU samples but not in LAB flies (Figure 2B; see details in Supplementary Tables S2A,B). Additionally, when total values were compared, five OTUs (*Enterobacter* [OTU2], *Wolbachia, Rheinheimera, Enterobacter* [OTU168] and *Citrobacter*) were represented in at least 40% of the analyzed samples (Figure 2C). This overall analysis showed two out of five OTUs (*Enterobacter* [OTU2] and *Wolbachia*) with a high percentage of read counts and the other three OTUs (*Rheinheimera, Enterobacter* [OTU168] and *Citrobacter*) were represented by a low percentage of read counts (Figure 2C and Table 1).

### Gut Bacterial Community and Sex

Statistical comparisons of samples grouped by sex evidenced that there are not significant differences in the gut bacteriome composition between females and males of *A. fraterculus* (UniFrac distance; PERMANOVA *p* > 0.05; Supplementary Figure S1). Males and females showed the same distribution of bacterial OTUs and abundance, with *Enterobacter* and *Wolbachia* observed in a high relative abundance (Supplementary Figure S2), and similar values of Chao1, Shannon and Simpson indices (Supplementary Figures S3A,B,D). In addition, non-significant differences were observed between sexes for evenness (Pielou index), phylogenetic diversity (Faith index), and dominance (Berger–Parker index) (Supplementary Figures S3C,E,F). Therefore, sex was not considered for further analyses performed in this study.

### Effect of the Feeding Status and Fly Origin on the Gut Bacteriome

**FIGURE 3:**
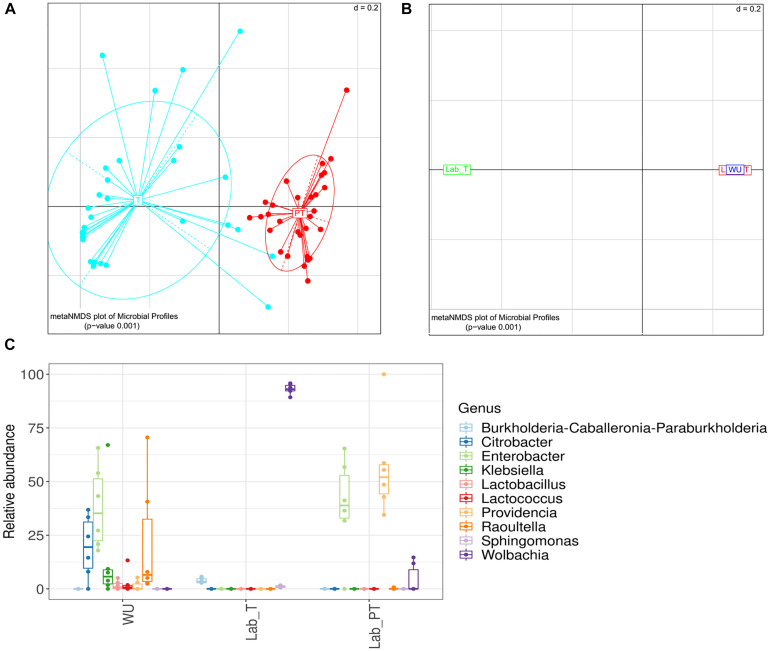
Effect of the feeding status and fruit fly origin on the gut bacteriome. **(A,B)** Meta-Non-metric Multidimensional scaling (meta NMDS) plot representing sample groups according to: **(A)** the feeding status (T, teneral; PT, post-teneral); **(B)** the origin (LAB, laboratory individuals with different feeding status [T, teneral; PT, post-teneral]; WU, wild individuals from ubajay with unknown feeding status). Significance *p-*value from PERMANOVA analysis; *d* = 0.2. **(C)** Relative abundance of bacterial genera associated to the digestive tract of flies from two different environmental origins (Lab, laboratory samples with different feeding status (T and PT); WU, wild samples from ubajay. Error bars correspond to standard error of the mean.

Significant differences in gut bacterial composition were observed between individuals with different feeding status (teneral [T] and post-teneral [PT]) (UniFrac distance; PERMANOVA *p* < 0.05; Figure 3A). In addition, we observed a differential distribution of bacterial OTUs and abundance between T and PT samples. *Wolbachia* was detected at a high prevalence (60.0% of reads) in T samples, whereas, *Enterobacter* (42.0% of reads), *Providencia* (18.3% of reads) and *Aeromonas* (15.2% of reads) dominated the gut bacteriome of PT individuals (Supplementary Figure S4). Furthermore, differential values of Chao1 and Simpson indices were observed between T and PT samples, showing higher values in T flies (Supplementary Figure S5). Additionally, evenness (Pielou index), phylogenetic diversity (Faith index), and dominance (Berger–Parker index) evidenced the same trends of significant differences between T and PT flies (Supplementary Figures S5C,E,F). Conversely, Shannon index showed no significant differences between T and PT flies (Supplementary Figure S5B).

In relation to the fly origin and feeding status, significant differences were observed among gut samples from the laboratory (LAB_T and LAB_PT), and wild flies (WU) collected in ubajay trees with unknown feeding status (UniFrac distance; PERMANOVA *p* < 0.05; Figure 3B). The gut bacteriome of WU samples was dominated by *Enterobacter* OTU2 (37.86% of reads), *Raoultella* (21.75% of reads) and *Citrobacter* (19.50% of reads) (Supplementary Table S2B) whereas LAB_PT gut bacteria was mainly represented by *Enterobacter* OTU2 (39.02%) and *Providencia* (56.09%) (Supplementary Table S2C). LAB_T gut community was dominated by *Wolbachia* (93.12% of reads) (Figure 3C).

**FIGURE 4:**
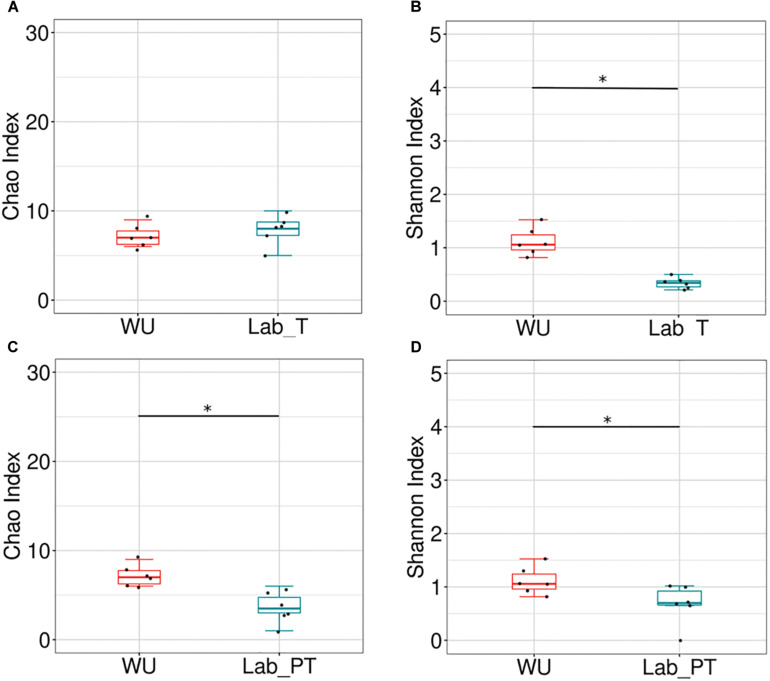
Effect of the fruit fly origin and feeding status on the gut bacterial community. Box plots representing bacterial richness and diversity among origins. Chao index **(A,C)** and Shannon index **(B,D)** in teneral (T) and post-teneral (PT) individuals. WU, wild individuals from ubajay with unknown feeding status; Lab, individuals from the laboratory colony. Dots indicate observed values and box plots depict means and standard deviation of the data. Bars with asterisks above boxes indicate significant *p-*values (paired comparisons, Kruskal–Wallis test).

Bacterial richness and diversity of teneral (T) and post-teneral (PT) flies from LAB, and WU showed significant differences in paired comparisons. Chao1 index and phylogenetic diversity (Faith index) showed non-significant differences between Lab_T and WU flies (Figure 4A and Supplementary Figure S6D). However, WU showed significantly higher values of Shannon index than LAB_T (Figure 4B). The same statistical differences were also observed for the Simpson index and evenness (Pielou index) (Supplementary Figures S6A,B). Congruently, Lab_T showed significantly higher values of dominance (Berger–Parker index) than WU (Supplementary Figure S6C).

In Lab_PT vs. WU comparisons, WU samples evidenced significantly higher values of Chao1, Shannon indices and phylogenetic diversity (Faith index) (Figures 4C,D and Supplementary Figure S6H). Conversely, additional analyses of diversity (Simpson index), evenness (Pielou index), and dominance (Berger–Parker index) showed non-significant differences between compared samples (Supplementary Figures S6E–G).

### Changes in Gut Bacteriome During Laboratory Colonization

**FIGURE 5:**
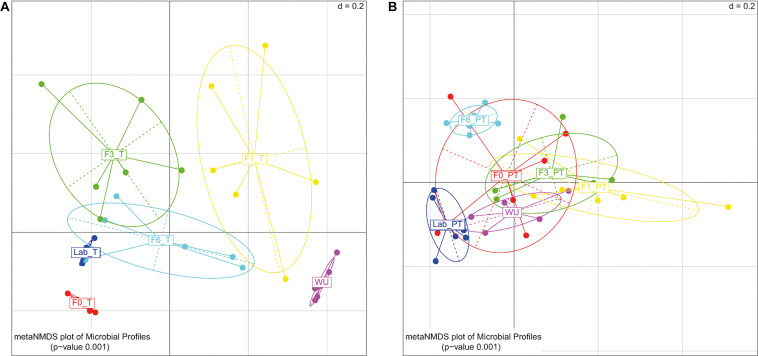
Gut-associated bacterial communities of *A. fraterculus* sp. 1 during laboratory colonization. Meta-Non-metric Multidimensional scaling (metaNMDS) plot of bacterial profile representing samples grouped by generation under artificial rearing (F0, F1, F3, and F6) and feeding status. **(A)** Teneral (T) samples. **(B)** Post teneral (PT) samples. WU, wild individuals from ubajay with unknown feeding status; Lab, individuals from the laboratory colony. Significance *p-*value from PERMANOVA analysis (*d* = 0.2).

Significant differences in the gut bacterial profile were observed across generations under artificial rearing conditions (F0–F6) both in teneral (T) and post-teneral (PT) flies (UniFrac distance; PERMANOVA *p* < 0.05; Figures 5A,B). Considering T flies from F0–F6, we observed that bacterial profiles differed between generations, and in all cases, were different from LAB_T and WU flies (PERMANOVA; *p* < 0.05; Table 2 and Figure 5A). For PT flies, F0–F6 generations differed in paired comparisons between them and among LAB_PT, and WU. Interestingly, bacterial profiles of teneral and post-teneral F6 were significantly different from teneral and post-teneral LAB samples, respectively (Table 2 and Figures 5A,B).

**TABLE 2:** PERMANOVA analysis – pair-wise comparisons.

Comparison	*p*-Value	Corr. *p*-value
F0_T-F1_T	0.005	0.006
F0_T-F3_T	0.001	0.003
F0_T-F6_T	0.003	0.0045
F0_T-LAB_T	0.003	0.005
F0_T-WU_unk	0.003	0.0045
F1_T-F3_T	0.005	0.0057
F1_T-F6_T	0.009	0.010
F1_T-LAB_T	0.002	0.006
F1_T-WU_unk	0.005	0.00625
F3_T-F6_T	0.002	0.021
F3_T-LAB_T	0.004	0.005
F3_T-WU_unk	0.004	0.006
F6_T-LAB_T	0.01	0.011
F6_T-WU_unk	0.004	0.0066
LAB_T-WU_unk	0.001	0.006
F0_PT-F1_PT	0.017	0.024
F0_PT-F3_PT	0.021	0.024
F0_PT-F6_PT	0.017	0.0225
F0_PT-LAB_PT	0.004	0.008
F0_PT-WU_unk	0.019	0.023
F1_PT-F3_PT	0.053	0.053
F1_PT-F6_PT	0.005	0.0125
F1_PT-LAB_PT	0.001	0.0085
F1_PT-WU_unk	0.007	0.011
F3_PT-F6_PT	0.002	0.0064
F3_PT-LAB_PT	0.004	0.0085
F3_PT-WU_unk	0.004	0.0428
F6_PT-LAB_PT	0.002	0.01
F6_PT-WU_unk	0.003	0.0056
LAB_PT-WU_unk	0.004	0.012
F0_T-F1_T	0.005	0.006
F0_T-F3_T	0.001	0.003
F0_T-F6_T	0.003	0.0045
F0_T-LAB_T	0.003	0.005

**FIGURE 6:**
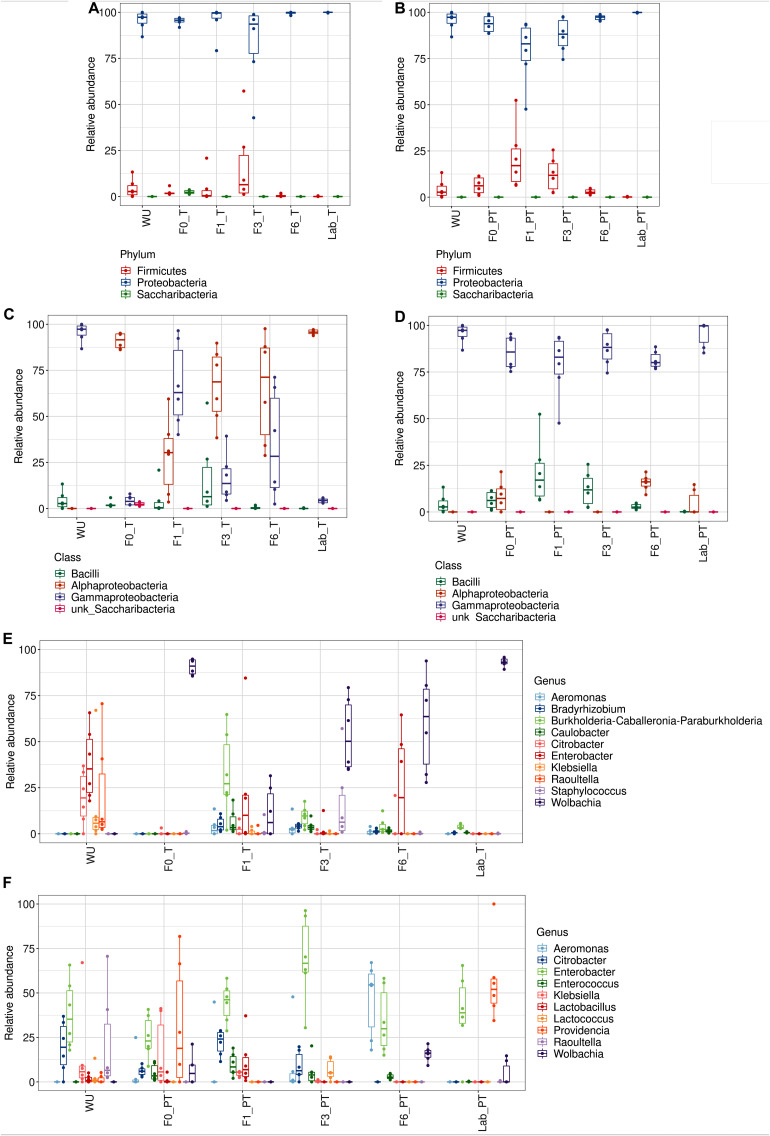
Gut-associated bacterial communities during of *A. fraterculus* sp. 1 laboratory colonization and feeding status. Relative abundance of the ten main OTUs identified. Taxonomic identification at the Phylum, Class and Genus levels for teneral (T) (**A–C**, respectively) and post-teneral (PT) (**D–F**, respectively) flies from F0, F1, F3, F6 generations under laboratory conditions. WU, wild individuals from ubajay with unknown feeding status; Lab, individuals from the laboratory colony.

The gut bacteriome associated with T flies was dominated by *Wolbachia* sp. (>50% of the total reads, found in all samples), with the exception of F1_T, in which, members of *Burkholderia–Caballeronia–Paraburkholderia* (OTU9) were the most dominant taxon (32.2% of reads) (Figures 6A–C and Supplementary Table S2C). Gammaproteobacteria was the most representative class in PT samples (>75% of reads, found in all samples) (Figures 6D,E). The same taxonomic classes with a differential relative abundance were observed in F0–F6_PT flies compared to F0–F6_T flies (Figures 6B,E). At the genus level, *Wolbachia* was detected in a low relative abundance in PT flies (<15%, detected in 50% of the samples) (Supplementary Tables S2C,D). Significant differences were observed when the *Wolbachia* relative abundance was compared between T and PT flies in each generation (Wilcoxon Rank Sum Test; *p* < 0.05), except for F1_T vs F1_PT (Supplementary Table S4). In addition, *Wolbachia* was not detected in WU samples (Figure 6F and Supplementary Tables S2A,B).

*Klebsiella* sp. and *Providencia* were noticeably abundant in F0_PT (>20%). In addition, we detected *Citrobacter* highly represented in WU, which was also found in F1_T and F6_T flies and in PT samples from F0, F1, and F3.

**FIGURE 7:**
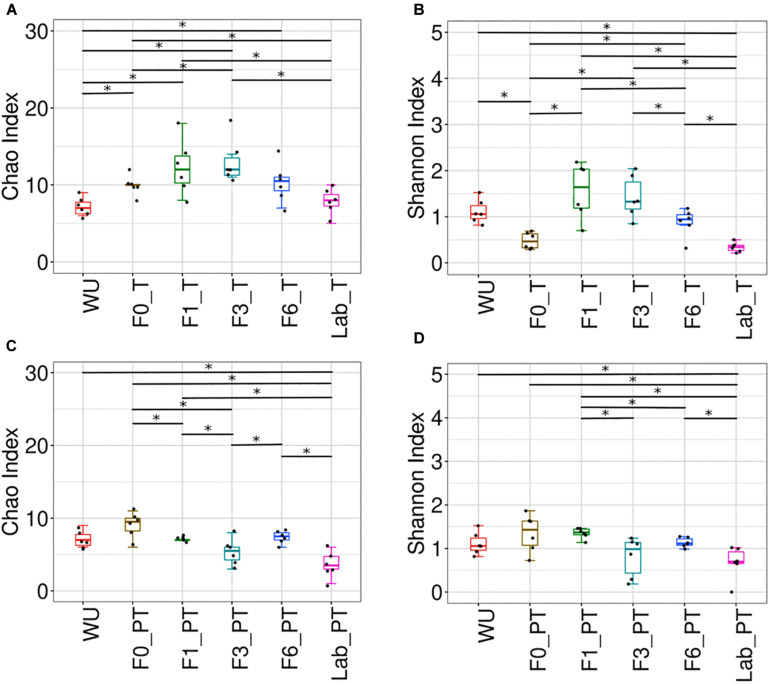
Bacterial community of the *A. fraterculus* sp. 1 digestive tract and laboratory colonization process. Bacterial richness and diversity associated to the digestive tract of F0–F6 individuals and laboratory flies. Chao index **(A,C)** and Shannon index **(B,D)** in teneral (T) and post-teneral (PT) individuals. WU, wild individuals from ubajay with unknown feeding status; Lab, individuals from the laboratory colony. See Figure 4 for dots and box plots description. Bars with asterisks above boxes indicate significant *p-*values (paired comparisons, Kruskal–Wallis test).

Teneral (T) and post-teneral (PT) flies presented different patterns of Chao1, Shannon and Simpson indices across the laboratory colonization process. For T flies, F1 and F3 showed the highest Chao1, Shannon and Simpson values (Figures 7A,B, Supplementary Table S3, and Supplementary Figure S7A) and F3 resulted significantly different from F0 and LAB (for the three indices, Figures 7A,B and Supplementary Figure S7A). In addition, LAB_T showed significant differences in Chao1 index compared to F0, F1 and F3. LAB_T and WU showed the lowest values for this index. In the case of Shannon and Simpson indices analysis, differentiation between paired comparisons of LAB_T and F1–F6_T flies and LAB_T-WU was detected. LAB_T and F0_T flies displayed the lowest Shannon index values but non-significant differences were detected between them (Figure 7B and Supplementary Figure S7A). With regard to PT flies, significant differences were observed between LAB_PT and WU, and each paired comparisons of LAB_PT with F0, F1, and F6 PT for Chao1 index. Differential values were also observed in F0–F1, F0–F3, F1–F3, and F3–F6 comparisons for this parameter. In sum, T flies showed a tendency to a reduction of Chao1, Shannon, Simpson, Faith and Pielou indices, as generations under artificial rearing increased (F1–F6), congruently with an increase of dominance estimated by Berger–Parker index (Figures 7A,B and Supplementary Figures S7B–D). Laboratory flies showed the lowest values for Shannon, Simpson, Chao1, phylogenetic diversity (Faith) and evenness (Pielou) estimators together with the highest values recorded for Berger–Parker index. This tendency was not detected in PT flies (Figures 7C,D and Supplementary Figure S7).

## Discussion

In the present work, we analyzed the bacterial community associated with the digestive tract of *A. fraterculus* sp. 1 adults using 16S *rRNA* amplicon sequencing. We were able to identify 32 OTUs in this gut bacteriome. From these classified taxonomic units, 29 OTUs were described at the genus level, one OTU (OTU19) remains unclassified under phylum level, identified as unknown Saccharibacteria and two other OTUs (15 and 124) were placed in two distinct taxonomic positions, showing lower similarity than 97% with *Raoultella* sp. and *Klebsiella* sp., respectively.

Overall analyses suggested that the gut bacterial profile was dominated by Proteobacteria, particularly by Alpha and Gammaproteobacteria; followed by Firmicutes and Saccharibacteria. Within the Gammaproteobacteria, the Enterobacteriaceae family was dominant in the gut of *A. fraterculus*, in agreement with previous reports in Tephritidae fruit flies (Behar et al., 2008; Jurkevitch, 2011; Müller, 2013; Morrow et al., 2015; Ventura et al., 2018; Augustinos et al., 2019; Koskinioti et al., 2019). The most abundant genera, were *Wolbachia* (Alphaproteobacteria) and *Enterobacter* (Gammaproteobacteria), followed by other members of the Gammaproteobacteria such as *Providencia*, *Aeromonas*, *Citrobacter, Burkholderia–Caballeronia–Paraburkholderia, Klebsiella* and *Raoultella.* Tephritid literature reports these genera of Proteobacteria with specific functions: *Wolbachia* with cytoplasmic incompatibility and male killing (Boller and Bush, 1974; Riegler and Stauffer, 2002; Zabalou et al., 2004, 2009; Apostolaki et al., 2011; Conte et al., 2019; Devescovi et al., 2019; Mateos et al., 2020), *Enterobacter*, *Citrobacter*, *Burkholderia*, *Klebsiella* and *Raoultella* with nitrogen metabolism (Murphy et al., 1988; Behar et al., 2005; Raza et al., 2020), and *Providencia* with pathogenic effects (Msaad Guerfali et al., 2018; Ksentini et al., 2019). In addition, *Acinetobacter* and *Rheinheimera* (Gammaproteobacteria) were detected in low abundance (0,1% of total reads) but in a high percentage of samples (33.33 and 46.97% of total samples, respectively) and wide distribution (Lab, WU, F0, F1–F6 flies). Despite *Acinetobacter* having been reported in several studies on fruit flies, its role is so far unclear (Kounatidis et al., 2009; Deutscher et al., 2019).

The gut bacteriome of *Anastrepha* genus was previously addressed using different tools. In *A. ludens*, *A. obliqua*, *A. serpentina*, and *Anastrepha striata*, *Escherichia* was one of the dominant genera (Ventura et al., 2018). Nonetheless, *Escherichia* was not identified in our samples, nor in *A. grandis*, *A. ludens*, and two morphotypes of *A. fraterculus* studied by Augustinos et al. (2019). For *A. fraterculus*, Proteobacteria dominate the gut bacterial community according to Augustinos et al. (2019) and our results. However, Müller (2013) found that the gut bacteriome of a Brazilian wild population of *A. fraterculus* was dominated by *Actinobacteria*. This author targeted a different region of the 16S *rRNA* gene, which could partially explain the differences; however, the main explanation is probably associated to environmental variation (Augustinos et al., 2019).

Despite some differences in the three studies on *A. fraterculus*, a rather clear pattern seems to emerge with Enterobacteriaceae being consistently the most abundant family associated to *A. fraterculus*, and particularly *Enterobacter* as the genus with the highest abundance. Recent studies have revealed the importance of *Enterobacter* spp. associated to Tephritidae (Kyritsis et al., 2017, 2019; Noman et al., 2020). As an example of essential contribution of this symbiont to fruit fly physiology, multiple traits have been addressed, including: nitrogen fixation and pectinolytic activity (Behar et al., 2005; Aharon et al., 2012) as well as, provision of essential and non-essential amino acids and vitamins (Azis et al., 2019) and its role on host behavior and fitness (Hamden et al., 2013; Augustinos et al., 2015; Kyritsis et al., 2017, 2019; Raza et al., 2020). These beneficial effects might explain why adding *Enterobacter* spp. to the larval diet in *C. capitata* improved pupal and adult productivity, as well as a faster development, particularly of males (Hamden et al., 2013; Augustinos et al., 2015). Further studies on the physiological role of *Enterobacter* spp. in *A. fraterculus* sp. 1 will bring valuable information to be applied to mass rearing protocols and environmentally safe control strategies.

Within Firmicutes, *Enterococcus* was the most abundant genus in the *A. fraterculus* gut bacterial community. A recent publication revealed that diet enriched with *Enterococcus* reduced the duration of the larval stage, increased pupal weight, and increased longevity in *Bactrocera dorsalis* (Khaeso et al., 2017). Finally, within the Saccharibacteria phylum, we detected an unclassified taxon that was exclusively found in F0_T flies. Similar results were observed by Koskinioti et al. (2019) who detected an unknown family of the order Saccharimonadales in the gut symbiotic communities of *Bactrocera oleae* wild samples.

Our results evidenced the presence of five common bacterial OTUs (*Enterobacter* [OTU2], *Providencia, Enterococcus, Rheinheimera* and *Acinetobacter* [OTU31]) described as the core bacteriome associated to the gut of *A. fraterculus* sp. 1 adult individuals. Digestive core bacteria and their contribution to the host biology have been addressed in other Tephritidae species including *C. capitata*, *B. minax*, and *Zeugodacus cucurbitae* (Wang et al., 2014; Yong et al., 2017; Andongma et al., 2019; reviewed by Deutscher et al., 2019). Particularly, our study showed an initial evaluation of the taxonomic composition of *A. fraterculus* sp. 1 core bacteria, with a restricted sampling considering different origins. In addition, we detected non-shared bacteria between laboratory and wild populations (WU, F0) and a population under adaptation (F1–F6), which could bring useful information to perform a further characterization and selection of bacterial isolates with potential benefits to improve mass rearing protocols and fitness of adults in the field in assistance to the SIT development against *A. fraterculus* sp. 1.

Changes in gut bacterial composition during laboratory colonization were strongly associated to the feeding status and generation. After six generations of laboratory rearing, the bacterial community is still changing and seems to maintain the differentiation from LAB flies. In our experiment, females from F0, F1, and F2 did not lay eggs in artificial OUs, and thus were offered mangoes. Artificial units were used from F3 onward. Our results are in line with previous studies conducted in other fruit fly species (Deutscher et al., 2019) and are strongly associated to the feeding behavior. Accordingly, the lack of differences in gut bacterial composition between females and males obtained in our work and in other related articles (Wang et al., 2014; Morrow et al., 2015; Augustinos et al., 2019) supported a substantial role of nutrition on the digestive microbiota composition, however, the bacterial diversity associated to different origins remained to be an important point of differentiation. These findings were also supported by studies performed in *B. oleae* (Koskinioti et al., 2019; revised by Deutscher et al., 2019).

We identified two OTUs (OTU22 assigned to *Commensalibacter* and OTU19, classified as an unknown member of the *Saccharibacteria* phylum) exclusively present in teneral wild flies from feijoa host fruit. These OTUs were no longer detected in F1 onward. Similarly, *Citrobacter* (OTU65) and *Klebsiella* (OTU124) were found in high abundance in wild adult flies (WU) and wild flies from feijoa (F0), but then slowly decreased as laboratory colonization progressed (F1 to F6); not being detected at all in LAB fruit fly guts. In other fruit fly species, *Citrobacter* [as all Enterobacteriaceae (Octavia and Lan, 2014)] has been shown to be capable of reducing nitrate to nitrite (Borenshtein and Schauer, 2006). Furthermore, the analysis of tryptic soy broth culture filtrates of *Citrobacter freundii* isolated from *A. ludens* showed that it contained greater amounts of nitrogenated compounds (Robacker and Bartelt, 1997). Because in nature nitrogen is a rather scarce resource (Mattson, 1980) and most tephritids need protein to achieve sexual maturation, gut bacteria may play a key role by making nitrogen available to their hosts. However, under laboratory conditions flies had unlimited access to a highly rich peptide source (hydrolyzed yeast) and the presence of some of these bacterial groups may no longer be essential. This might indicate that these OTUs are relevant for the host fitness in nature but are probably not needed under laboratory conditions. Alternatively, vertical transmission of these OTUs could be compromised under artificial rearing (Sacchetti et al., 2014; Augustinos et al., 2019; Deutscher et al., 2019). Under this scenario, some gut bacteria might have disadvantageous conditions under the laboratory, and this situation could have negatively impacted on their diversity. However, more work is needed to address potential negative selection on specific bacterial groups as a consequence of drastic environmental changes, such as those suffered by wild flies when are brought to the laboratory and breed under captivity. Understanding the physiological role of these OTUs might shed light on important attributes of wild flies that are lost because of domestication.

Teneral flies of the F0 generation had the lowest values of diversity estimated through different indices among F0–F6 generations; whereas F1 and F3 showed the highest values for these parameters. This may be related with stochastic drift processes emerging after disturbs and associated to environmental factors that may have compromised the dominance of *Wolbachia* at least in F1, right after the introduction of the host to novel conditions (Staubach et al., 2013). After F3, and in F6 and LAB teneral flies, *Wolbachia* dominated the gut bacteria community. On the other hand, in post-teneral flies (15-day-old, fed individuals) the community is dominated by Enterobacter. This might indicate that the community is stabilized in post-teneral flies, and this status would be less exposed to environmental stressors. In agreement with our results, a recent study performed in *A. obliqua* (whole body) showed *Wolbachia* sp. more abundant in larvae and *Enterobacter* sp. in adults (Gallo-Franco and Toro-Perea, 2020).

Changes in the composition of digestive bacteria during the domestication process could affect the physiology and behavior of the host eventually leading to limitations in the context of the SIT, which requires a productive mass rearing but also sexually competitive. Previous studies in *C. capitata* highlighted the contribution of gut bacterial symbionts associated to the digestive tract to male sexual performance, flight ability and longevity under starvation and enhancement of the SIT (Niyazi et al., 2004; Ben-Yosef et al., 2008a; Ben-Ami et al., 2010; Gavriel et al., 2011; Augustinos et al., 2015; Kyritsis et al., 2017). Understanding the replacement of gut bacteria associated with domestication is an initial step to determine which bacterial symbionts are the most affected. This characterization would allow to design domestication protocols that either maintain key players in the gut of domesticated insects or restore them as part of the rearing process (i.e., use specific bacteria as dietary probiotics). Because the insect gut microbiome includes not only the gut bacteriome, but also virus, protozoa, fungi, yeasts that might interact not only with the host but also among them (Gurung et al., 2019), future studies should aim at disentangling these complex interactions and enrich our understanding of the physiology and behavior of this important fruit pest of South America.

## Conclusion

Our work revealed that the origin and feeding status could shape the gut bacterial community of *A. fraterculus* adults. We observed a dynamic interaction between *A. fraterculus* and its microbiota. Upon emergence, the gut is dominated by *Wolbachia* but as flies feed and age, other genera such as *Enterobacter*, *Providencia*, and *Citrobacter* become more abundant, and the whole community more diverse, reaching a seemingly stable composition. We evidenced gradual changes during the first steps of the laboratory colonization process keeping, however, a degree of differentiation between flies under adaptation and a well-established laboratory strain. The taxonomic identification of the gut bacterial community of *A. fraterculus* sp. 1 from Argentina and the analysis of key factors modeling the structure and composition of the gut bacteriome provide valuable and novel information. Understanding the dynamic interaction between a tephritid host and digestive bacterial symbionts will enable to improve environmentally safe control strategies against fruit fly pest species.

## Data Availability Statement

The datasets presented in this study can be found in online repositories. 16S *rRNA* gene sequences reported in this study have been deposited in the NCBI under BioProject number PRJNA624812.

## Author Contributions

SL, LP, GT, KB, and DS conceived and designed the study. JS, LP, FM, RR, EA, and PS conducted the experiments. JS, DS, SL, and GT analyzed the results. JS, DS, SL, KB, JC, and GT drafted the manuscript. All authors reviewed the manuscript.

## Conflict of Interest

The authors declare that the research was conducted in the absence of any commercial or financial relationships that could be construed as a potential conflict of interest.

## Supplementary Material

Supplementary Figure 1 *A. fraterculus* sp. 1 digestive bacterial community and sex. Meta-Non-metric Multidimensional scaling (metaNMDS) plot of bacterial profile representing samples grouped by sex: male in blue; female in red color. PERMANOVA analysis *p*-value (*d* = 0.2).

Supplementary Figure 2 Relative abundance of OTUs (at genus level) of bacterial community associated to the digestive tract of flies grouped by sex.

Supplementary Figure 3 Bacterial diversity found in *A. fraterculus* sp. 1 digestive tract of adult individuals grouped by sex. **(A)** Chao index. **(B)** Shannon index. **(C)** Faith index. **(D)** Simpson index. **(E)** Pielou index and **(F)** Berger index. See Figure 4 for dots and box plots description.

Supplementary Figure 4 Relative abundance of OTUs (at genus level) of bacterial community associated to the digestive tract of flies grouped by feeding status: T (teneral); PT (post-teneral).

Supplementary Figure 5 Bacterial diversity found in *A. fraterculus* sp. 1 digestive tract of adult individuals grouped by feeding status: T (teneral); PT (post-teneral). **(A)** Chao index. **(B)** Shannon index. **(C)** Faith index. **(D)** Simpson index. **(E)** Pielou index and **(F)** Berger index. See Figure 4 for dots and box plots description. Bars with asterisks above boxes indicate significant *p-*values (paired comparisons, Kruskal–Wallis test).

Supplementary Figure 6 Bacterial diversity among origins - additional parameters. Simpson index **(A,E)**; Pielou index **(B,F)**; Berger-Parker index **(C,G)**; and Faith index **(D,H)** in teneral (T) and post-teneral (PT) individuals. WU, wild individuals from ubajay with unknown feeding status; Lab, individuals from the laboratory colony. Dots indicate observed values and box plots depict means and standard deviation of the data. Bars with asterisks above boxes indicate significant *p-*values (paired comparisons, Kruskal–Wallis test).

Supplementary Figure 7 Bacterial diversity during laboratory colonization - additional parameters. Simpson index **(A,E)**; Pielou index **(B,F)**; Berger–Parker index **(C,G)**; and Faith index **(D,H)** in teneral (T) and post-teneral (PT) individuals. WU, wild individuals from ubajay with unknown feeding status; Lab, individuals from the laboratory colony. See Figure 4 for dots and box plots description. Bars with asterisks above boxes indicate significant *p-*values (paired comparisons, Kruskal–Wallis test).

Supplementary Table 1 Identification of gut samples and description of rearing conditions of *A. fraterculus* sp. 1 laboratory colonies and wild flies used in this work. F0–F6 indicates samples from generations under laboratory colonization. Lab, laboratory samples; WU, wild samples from ubajay; T, teneral feeding status; PT, post-teneral feeding status; M, male; F, female; Unk, unknown, without information on age, feeding status, and diet (at larval and adult stages).

Supplementary Table 2 OTU description and representation in *A. fraterculus* sp. 1 gut bacteriome. **(A)** OTU representation – Percentage of samples. **(B)** OTU representation – Percentage of read counts. **(C)** OTU representation – Percentage of read counts considering feeding status. **(D)** OTU representation in % of samples considering feeding status. OTUs belonging to the bacterial core proposed are highlighted.

Supplementary Table 3 Bacterial diversity and richness (Shannon and Chao indices).

Supplementary Table 4 Relative abundance of *Wolbachia* (OTU 1) pair-wise comparisons (teneral [T] vs post-teneral [PT]) using Wilcoxon Rank Sum Test. Group compared: generation F0–F6 and laboratory flies (Lab). Mean values of each compared group were considered to the statistical analysis.
