# Supplementary figures and images for: Gut Bacteriome Analysis of Anastrepha fraterculus sp. 1 During the Early Steps of Laboratory Colonization
Source: Front Microbiol. 2020 Oct 20;11:570960. doi: 10.3389/fmicb.2020.570960 (PMC7606190; doi:10.3389/fmicb.2020.570960)

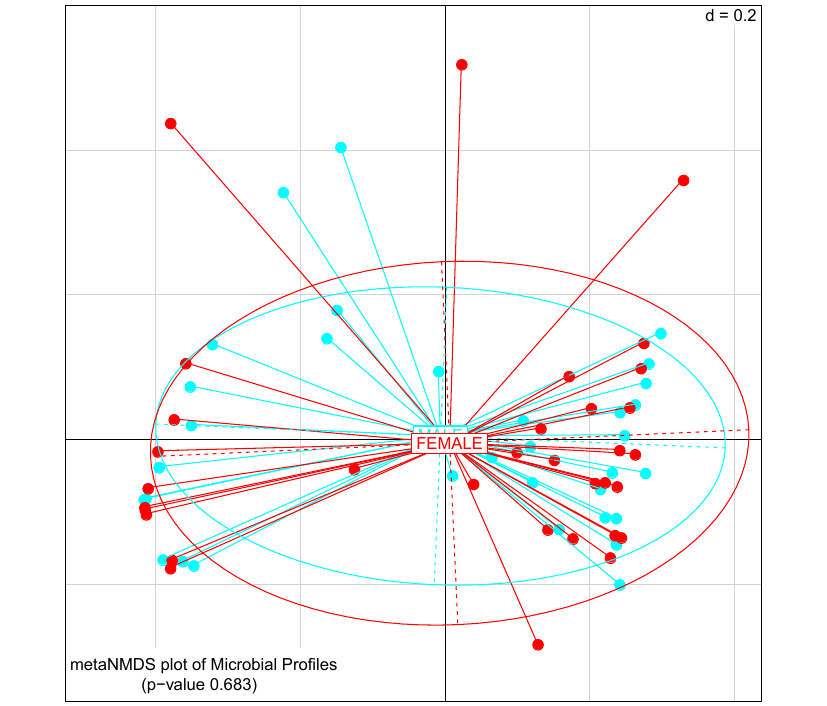

Supplement: Supplementary Figure 1 — A. fraterculus sp. 1 digestive bacterial community and sex. Meta-Non-metric Multidimensional scaling (metaNMDS) plot of bacterial profile representing samples grouped by sex: male in blue; female in red color. PERMANOVA analysis p-value (d = 0.2). [file Image_1.TIF]

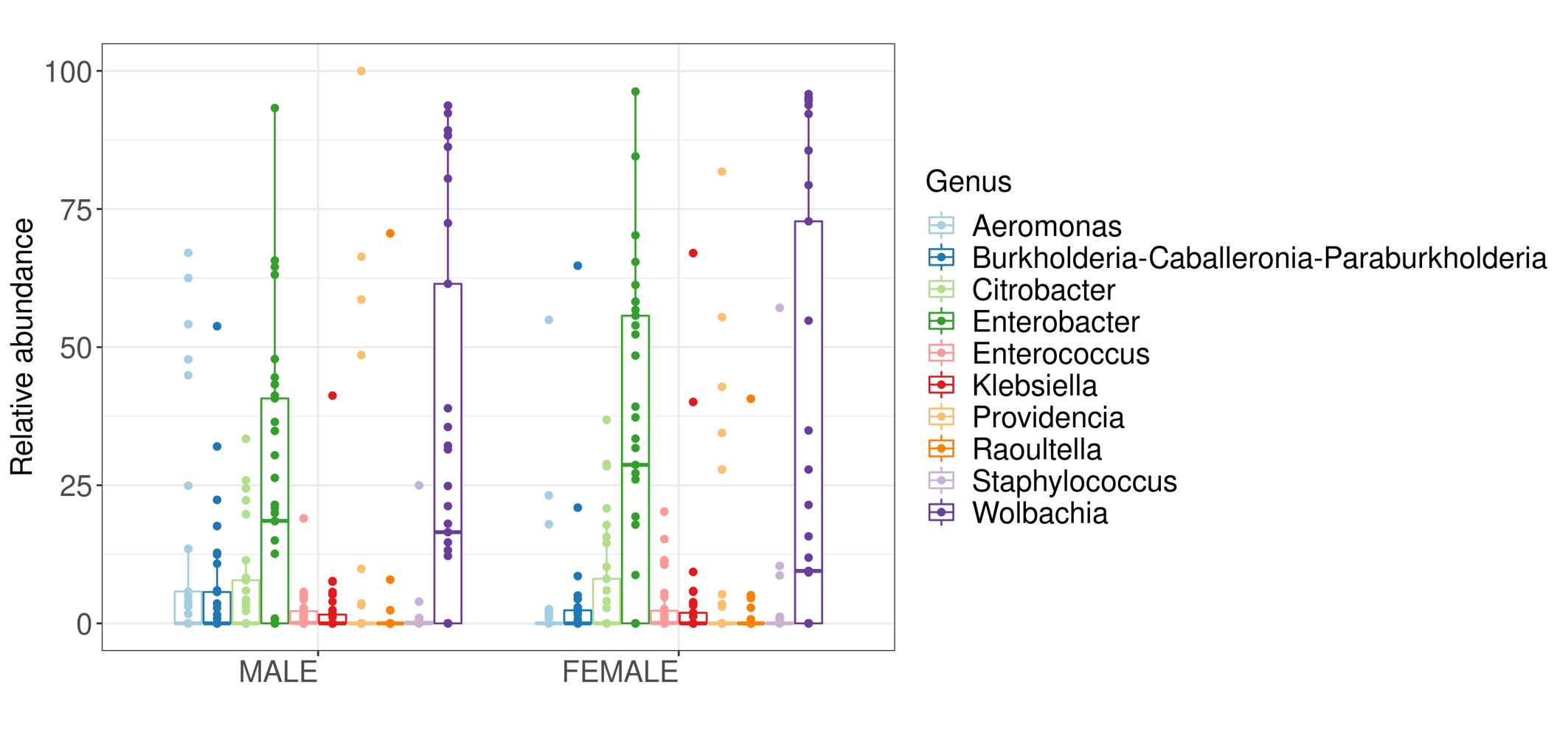

Supplement: Supplementary Figure 2 — Relative abundance of OTUs (at genus level) of bacterial community associated to the digestive tract of flies grouped by sex. [file Image_2.TIF]

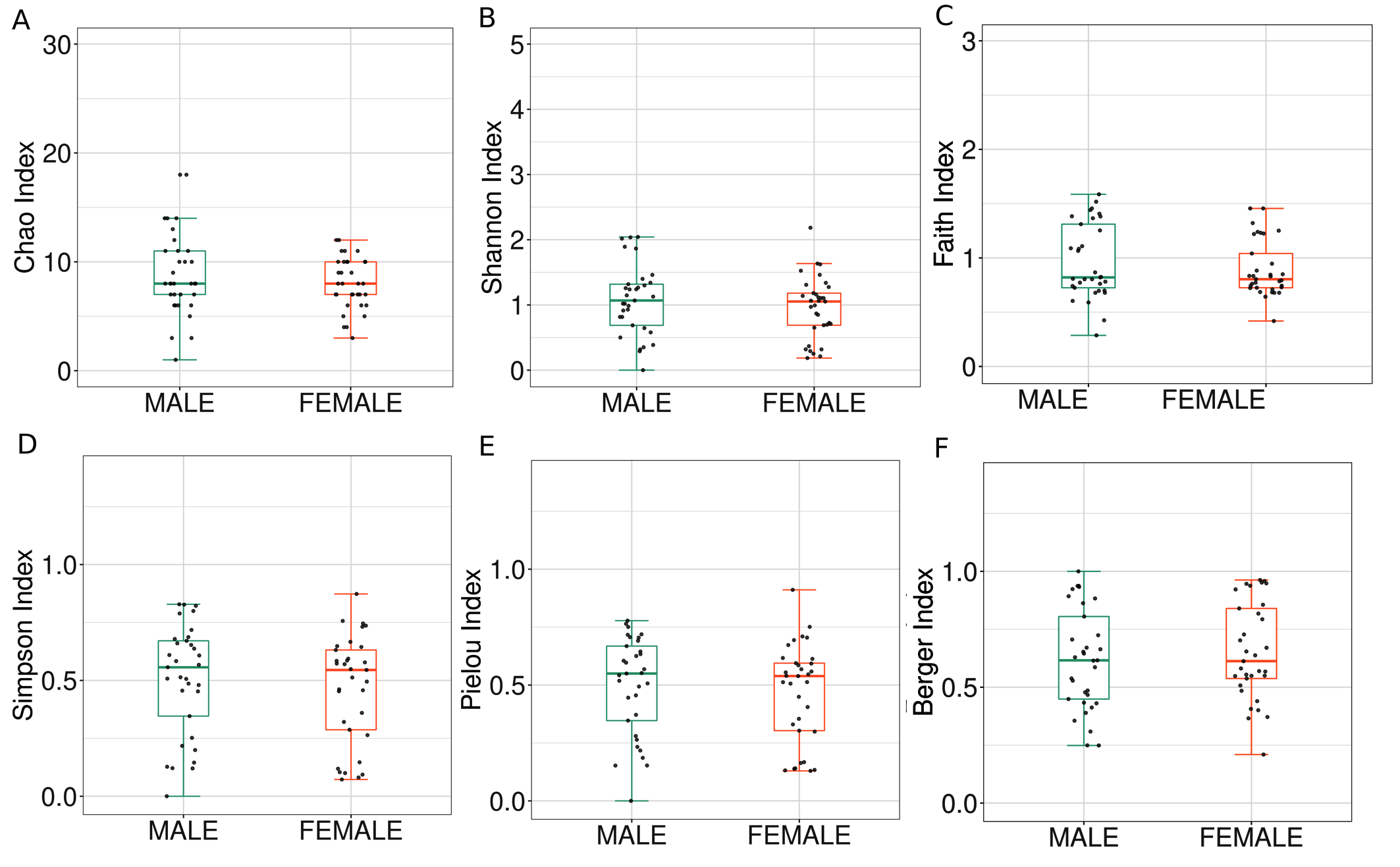

Supplement: Supplementary Figure 3 — Bacterial diversity found in A. fraterculus sp. 1 digestive tract of adult individuals grouped by sex. (A) Chao index. (B) Shannon index. (C) Faith index. (D) Simpson index. (E) Pielou index and (F) Berger index. See Figure 4 for dots and box plots description. [file Image_3.TIF]

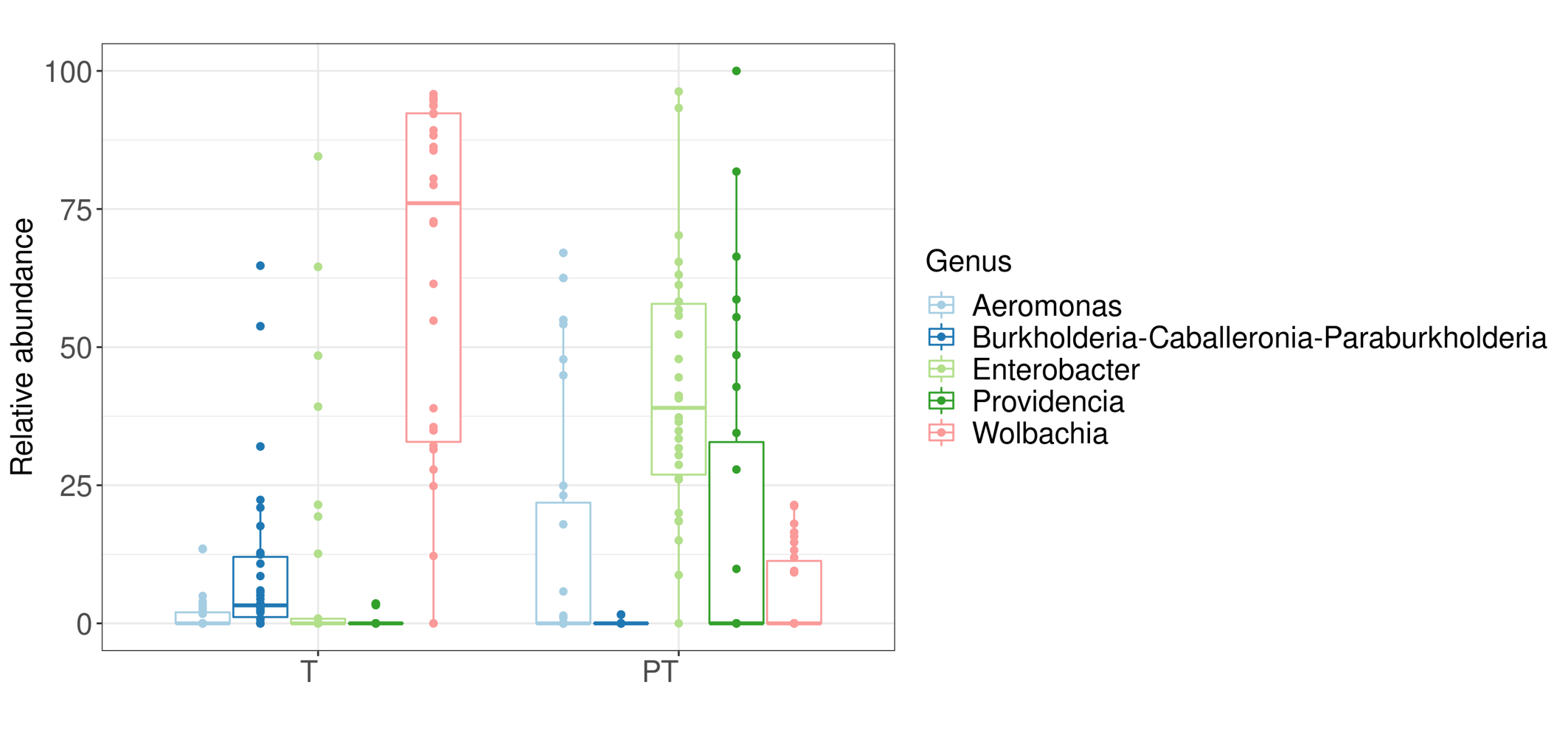

Supplement: Supplementary Figure 4 — Relative abundance of OTUs (at genus level) of bacterial community associated to the digestive tract of flies grouped by feeding status: T (teneral); PT (post-teneral). [file Image_4.TIF]

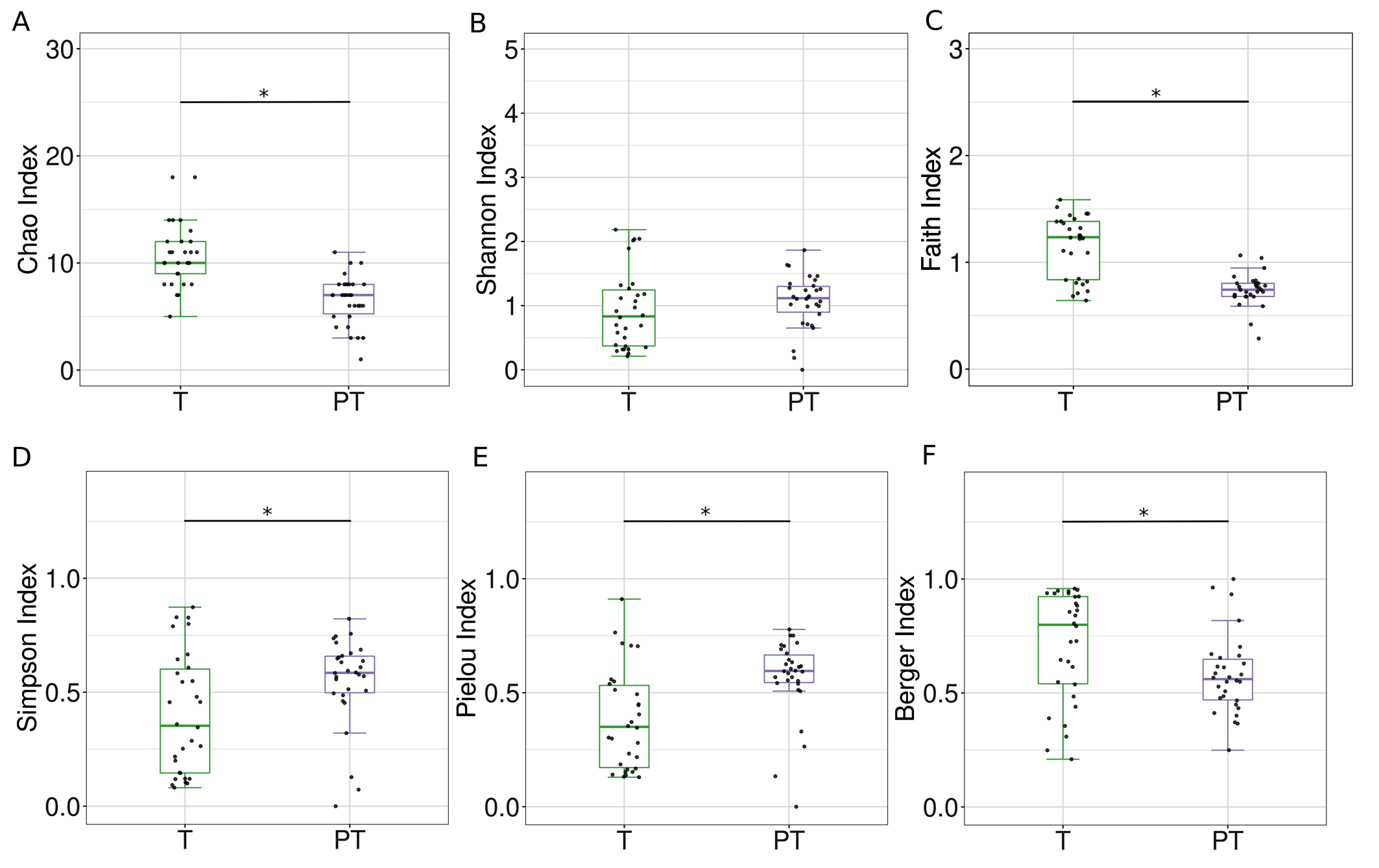

Supplement: Supplementary Figure 5 — Bacterial diversity found in A. fraterculus sp. 1 digestive tract of adult individuals grouped by feeding status: T (teneral); PT (post-teneral). (A) Chao index. (B) Shannon index. (C) Faith index. (D) Simpson index. (E) Pielou index and (F) Berger index. See Figure 4 for dots and box plots description. Bars with asterisks above boxes indicate significant p-values (paired comparisons, Kruskal–Wallis test). [file Image_5.TIF]

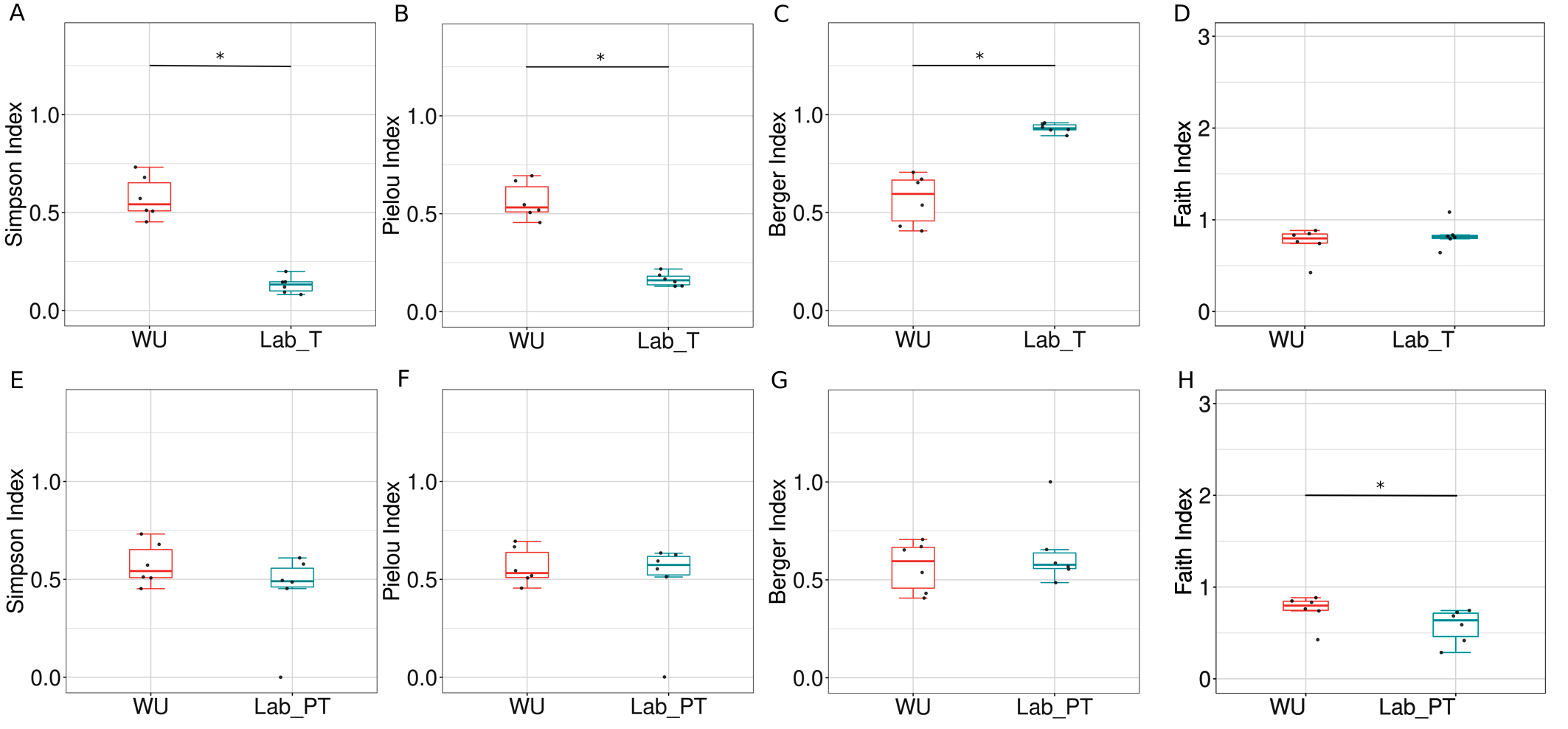

Supplement: Supplementary Figure 6 — Bacterial diversity among origins - additional parameters. Simpson index (A,E); Pielou index (B,F); Berger-Parker index (C,G); and Faith index (D,H) in teneral (T) and post-teneral (PT) individuals. WU, wild individuals from ubajay with unknown feeding status; Lab, individuals from the laboratory colony. Dots indicate observed values and box plots depict means and standard deviation of the data. Bars with asterisks above boxes indicate significant p-values (paired comparisons, Kruskal–Wallis test). [file Image_6.TIF]

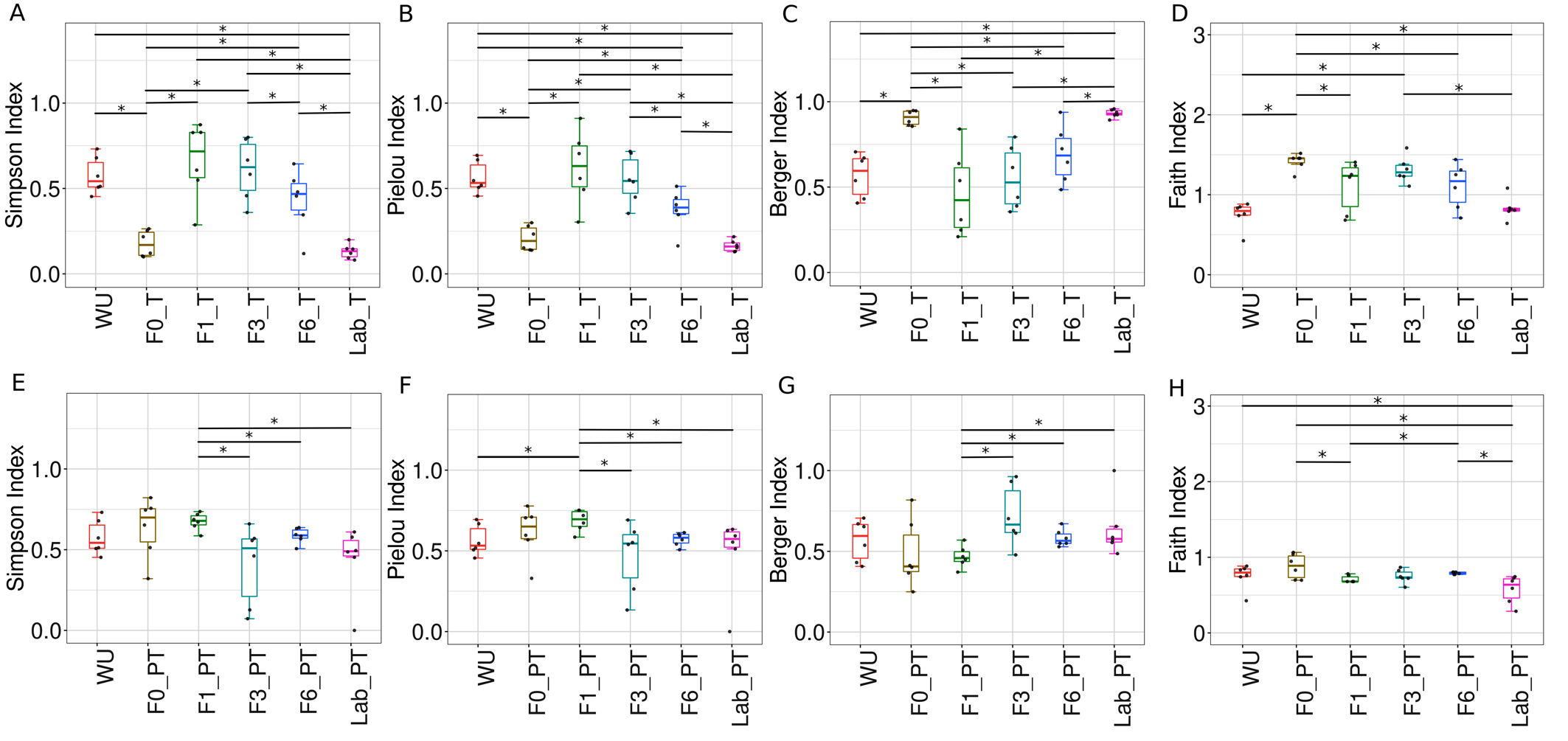

Supplement: Supplementary Figure 7 — Bacterial diversity during laboratory colonization - additional parameters. Simpson index (A,E); Pielou index (B,F); Berger–Parker index (C,G); and Faith index (D,H) in teneral (T) and post-teneral (PT) individuals. WU, wild individuals from ubajay with unknown feeding status; Lab, individuals from the laboratory colony. See Figure 4 for dots and box plots description. Bars with asterisks above boxes indicate significant p-values (paired comparisons, Kruskal–Wallis test). [file Image_7.TIF]
