# Supplementary material for: Gut Bacteriome Analysis of Anastrepha fraterculus sp. 1 During the Early Steps of Laboratory Colonization
Source: Front Microbiol. 2020 Oct 20;11:570960. doi: 10.3389/fmicb.2020.570960 (PMC7606190; doi:10.3389/fmicb.2020.570960)
Supplement: Supplementary Table 1 — Identification of gut samples and description of rearing conditions of A. fraterculus sp. 1 laboratory colonies and wild flies used in this work. F0–F6 indicates samples from generations under laboratory colonization. Lab, laboratory samples; WU, wild samples from ubajay; T, teneral feeding status; PT, post-teneral feeding status; M, male; F, female; Unk, unknown, without information on age, feeding status, and diet (at larval and adult stages). [file Table_1.DOC]

| **S**ample ID | **Sample order ID** | **Sex** | **Age / Feeding status (days)** | **Larval diet** | **Adult diet** | **Oviposition Unit (OU) Type** | **Fruit exposition** |
| --- | --- | --- | --- | --- | --- | --- | --- |
|  |  |  |  |  |  |  |  |
|  |  |  |  |  |  |  |  |
| F0_T_F | 1 | Female | 1 / unfed | Feijoa | .. | .. | .. |
|  | 2 | Female | 1 / unfed | Feijoa | .. | .. | .. |
|  | 3 | Female | 1 / unfed | Feijoa | .. | .. | .. |
| F0_T_M | 4 | Male | 1 / unfed | Feijoa | .. | .. | .. |
|  | 5 | Male | 1 / unfed | Feijoa | .. | .. | .. |
|  | 6 | Male | 1 / unfed | Feijoa | .. | .. | .. |
| F0_PT_F | 7 | Female | 15 / fed | Feijoa | Artificial | Mango | Mango |
|  | 8 | Female | 15 / fed | Feijoa | Artificial | Mango | Mango |
|  | 9 | Female | 15 / fed | Feijoa | Artificial | Mango | Mango |
| F0_PT_M | 10 | Male | 15 / fed | Feijoa | Artificial | Mango | Mango |
|  | 11 | Male | 15 / fed | Feijoa | Artificial | Mango | Mango |
|  | 12 | Male | 15 / fed | Feijoa | Artificial | Mango | Mango |
| F1_T_F | 13 | Female | 1 / unfed | Mango | .. | .. | .. |
|  | 14 | Female | 1 / unfed | Mango | .. | .. | .. |
|  | 15 | Female | 1 / unfed | Mango | .. | .. | .. |
| F1_T_M | 16 | Male | 1 / unfed | Mango | .. | .. | .. |
|  | 17 | Male | 1 / unfed | Mango | .. | .. | .. |
|  | 18 | Male | 1 / unfed | Mango | .. | .. | .. |
| F1_PT_F | 19 | Female | 15 / fed | Mango | Artificial | Mango+Artificial | Mango |
|  | 20 | Female | 15 / fed | Mango | Artificial | Mango+Artificial | Mango |
|  | 21 | Female | 15 / fed | Mango | Artificial | Mango+Artificial | Mango |
| F1_PT_M | 22 | Male | 15 / fed | Mango | Artificial | Mango+Artificial | Mango |
|  | 23 | Male | 15 / fed | Mango | Artificial | Mango+Artificial | Mango |
|  | 24 | Male | 15 / fed | Mango | Artificial | Mango+Artificial | Mango |
| F3_T_F | 25 | Female | 1 / unfed | Artificial | .. | .. | .. |
|  | 26 | Female | 1 / unfed | Artificial | .. | .. | .. |
|  | 27 | Female | 1 / unfed | Artificial | .. | .. | .. |
| F3_T_M | 28 | Male | 1 / unfed | Artificial | .. | .. | .. |
|  | 29 | Male | 1 / unfed | Artificial | .. | .. | .. |
|  | 30 | Male | 1 / unfed | Artificial | .. | .. | .. |
| F3_PT_F | 31 | Female | 15 / fed | Artificial | Artificial | Artificial | Artificial |
|  | 32 | Female | 15 / fed | Artificial | Artificial | Artificial | Artificial |
|  | 33 | Female | 15 / fed | Artificial | Artificial | Artificial | Artificial |
| F3_PT_M | 34 | Male | 15 / fed | Artificial | Artificial | Artificial | Artificial |
|  | 35 | Male | 15 / fed | Artificial | Artificial | Artificial | Artificial |
|  | 36 | Male | 15 / fed | Artificial | Artificial | Artificial | Artificial |
| F6_T_F | 37 | Female | 1 / unfed | Artificial | .. | .. | .. |
|  | 38 | Female | 1 / unfed | Artificial | .. | .. | .. |
|  | 39 | Female | 1 / unfed | Artificial | .. | .. | .. |
| F6_T_M | 40 | Male | 1 / unfed | Artificial | .. | .. | .. |
|  | 41 | Male | 1 / unfed | Artificial | .. | .. | .. |
|  | 42 | Male | 1 / unfed | Artificial | .. | .. | .. |
| F6_PT_F | 43 | Female | 15 / fed | Artificial | Artificial | Artificial | Artificial |
|  | 44 | Female | 15 / fed | Artificial | Artificial | Artificial | Artificial |
|  | 45 | Female | 15 / fed | Artificial | Artificial | Artificial | Artificial |
| F6_PT_M | 46 | Male | 15 / fed | Artificial | Artificial | Artificial | Artificial |
|  | 47 | Male | 15 / fed | Artificial | Artificial | Artificial | Artificial |
|  | 48 | Male | 15 / fed | Artificial | Artificial | Artificial | Artificial |
| Lab_PT_F | 49 | Female | 15 / fed | Artificial | Artificial | Artificial | Artificial |
|  | 50 | Female | 15 / fed | Artificial | Artificial | Artificial | Artificial |
|  | 51 | Female | 15 / fed | Artificial | Artificial | Artificial | Artificial |
| Lab_PT_M | 52 | Male | 15 / fed | Artificial | Artificial | Artificial | Artificial |
|  | 53 | Male | 15 / fed | Artificial | Artificial | Artificial | Artificial |
|  | 54 | Male | 15 / fed | Artificial | Artificial | Artificial | Artificial |
| Lab_T_F | 55 | Female | 1 / unfed | Artificial | .. | .. | .. |
|  | 56 | Female | 1 / unfed | Artificial | .. | .. | .. |
|  | 57 | Female | 1 / unfed | Artificial | .. | .. | .. |
| Lab_T_M | 58 | Male | 1 / unfed | Artificial | .. | .. | .. |
|  | 59 | Male | 1 / unfed | Artificial | .. | .. | .. |
|  | 60 | Male | 1 / unfed | Artificial | .. | .. | .. |
| WU_unk_F | 61 | Female | unk | unk | unk | .. | .. |
|  | 62 | Female | unk | unk | unk | .. | .. |
|  | 63 | Female | unk | unk | unk | .. | .. |
| WU_unk_M | 64 | Male | unk | unk | unk | .. | .. |
|  | 65 | Male | unk | unk | unk | .. | .. |
|  | 66 | Male | unk | unk | unk | .. | .. |
