# Supplementary material for: Gut Bacteriome Analysis of Anastrepha fraterculus sp. 1 During the Early Steps of Laboratory Colonization
Source: Front Microbiol. 2020 Oct 20;11:570960. doi: 10.3389/fmicb.2020.570960 (PMC7606190; doi:10.3389/fmicb.2020.570960)
Supplement: Supplementary Table 2 — OTU description and representation in A. fraterculus sp. 1 gut bacteriome. (A) OTU representation – Percentage of samples. (B) OTU representation – Percentage of read counts. (C) OTU representation – Percentage of read counts considering feeding status. (D) OTU representation in % of samples considering feeding status. OTUs belonging to the bacterial core proposed are highlighted. [file Table_2.DOC]

**A.**

| **OTU ID** | **OTU (Genus)** | **# samples *** | **%**  **Samples **** | **OTU representation (% of samples)** | | | | | | |
| --- | --- | --- | --- | --- | --- | --- | --- | --- | --- | --- |
|  |  |  |  | **WU** | **F0** | **F1** | **F3** | **F6** | **F1-F6** | **LAB** |
| Otu18 | *Acinetobacter* | 13 | 19.70 | 0 | 50 | 8.33 | 16.66 | 8.33 | 11.11 | 25 |
| Otu31 | *Acinetobacter* | 22 | 33.33 | 16.67 | 50 | 25 | 41.66 | 16.66 | 27.78 | 41.66 |
| Otu10 | *Aeromonas* | 11 | 16.67 | 0 | 16.67 | 8.33 | 25 | 41.66 | 25.00 | 0 |
| Otu3 | *Aeromonas* | 14 | 21.21 | 0 | 0 | 25 | 33.33 | 58.33 | 38.89 | 0 |
| Otu13 | *Bradyrhizobium* | 24 | 36.36 | 0 | 8.33 | 50 | 50 | 50 | 50.00 | 41.66 |
| Otu6 | *Burkholderia-*  *Caballeronia-*  *Paraburkholderia* | 23 | 34.85 | 0 | 0 | 41.66 | 50 | 50 | 47.22 | 50 |
| Otu9 | *Burkholderia-*  *Caballeronia-*  *Paraburkholderia* | 6 | 9.09 | 0 | 16.67 | 33.33 | 0 | 0 | 11.11 | 0 |
| Otu8 | *Caulobacter* | 24 | 36.36 | 0 | 0 | 50 | 50 | 50 | 50.00 | 50 |
| Otu65 | *Citrobacter* | 27 | 40.91 | 83.33 | 58.33 | 66.66 | 50 | 8.33 | 41.67 | 0 |
| Otu22 | *Commensalibacter* | 3 | 4.55 | 0 | 25 | 0 | 0 | 0 | 0.00 | 0 |
| Otu168 | *Enterobacter* | 28 | 42.42 | 83.33 | 50 | 66.66 | 25 | 50 | 47.22 | 0 |
| Otu2 | *Enterobacter* | 42 | 63.64 | 100 | 50 | 75 | 58.33 | 75 | 69.44 | 41.66 |
| Otu52 | *Enterobacter* | 8 | 12.12 | 0 | 0 | 16.66 | 8.33 | 41.66 | 22.22 | 0 |
| Otu35 | *Enterococcus* | 19 | 28.79 | 16.67 | 66.67 | 50 | 0 | 0 | 16.67 | 33.33 |
| Otu5 | *Enterococcus* | 23 | 34.85 | 0 | 41.67 | 58.33 | 41.66 | 50 | 50.00 | 0 |
| Otu124 | *Klebsiella* | 20 | 30.30 | 83.33 | 33.33 | 66.66 | 25 | 0 | 30.56 | 0 |
| Otu11 | *Lactobacillus* | 11 | 16.67 | 50 | 16.67 | 50 | 0 | 0 | 16.67 | 0 |
| Otu12 | *Lactococcus* | 10 | 15.15 | 66.67 | 0 | 8.33 | 41.66 | 0 | 16.67 | 0 |
| Otu17 | *Mesorhizobium* | 19 | 28.79 | 0 | 0 | 33.33 | 50 | 50 | 44.44 | 25 |
| Otu21 | *Mesorhizobium* | 25 | 37.88 | 0 | 16.67 | 41.66 | 50 | 50 | 47.22 | 50 |
| Otu26 | *Neisseria* | 5 | 7.58 | 0 | 33.33 | 8.33 | 0 | 0 | 2.78 | 0 |
| Otu44 | *Neisseria* | 5 | 7.58 | 0 | 8.33 | 8.33 | 8.33 | 16.66 | 11.11 | 0 |
| Otu4 | *Providencia* | 15 | 22.73 | 33.33 | 41.67 | 8.33 | 8.33 | 0 | 5.56 | 50 |
| Otu15 | *Raoultella* | 8 | 12.12 | 100 | 0 | 8.33 | 0 | 0 | 2.78 | 8.33 |
| Otu136 | *Rheinheimera* | 31 | 46.97 | 83.33 | 41.67 | 25 | 41.66 | 75 | 47.22 | 33.33 |
| Otu16 | *Serratia* | 14 | 21.21 | 0 | 66.67 | 25 | 25 | 0 | 16.67 | 0 |
| Otu14 | *Sphingomonas* | 26 | 39.39 | 0 | 50.00 | 33.33 | 50 | 33.33 | 38.89 | 50 |
| Otu20 | *Staphylococcus* | 18 | 27.27 | 0 | 41.67 | 33.33 | 50 | 16.66 | 33.33 | 8.33 |
| Otu7 | *Staphylococcus* | 4 | 6.06 | 0 | 0 | 0 | 25 | 8.33 | 11.11 | 0 |
| Otu24 | *Streptococcus* | 17 | 25.76 | 0 | 41.67 | 25 | 41.66 | 33.33 | 33.33 | 0 |
| Otu19 | *Unknown Saccharibacteria* | 6 | 9.09 | 0 | 50 | 0 | 0 | 0 | 0.00 | 0 |
| Otu1 | *Wolbachia* | 38 | 57.58 | 0 | 75 | 25 | 50 | 100 | 58.33 | 66.66 |

*Number of *A. fraterculus* sp. 1 gut samples in which the OTU was detected.

**Percentage of *A. fraterculus* sp. 1 gut samples in which the OTU was detected in respect to the total of sequenced samples

**B.**

| **OTU ID** | **OTU ID**  **(Genus classification)** | **OTU representation (% of read counts)** | | | | | | |
| --- | --- | --- | --- | --- | --- | --- | --- | --- |
|  |  | **WU** | **F0** | **F1** | **F3** | **F6** | **F1-F6** | **LAB** |
| Otu18 | *Acinetobacter* | 0.00 | 0.67 | 0.02 | 0.06 | 0.01 | 0.03 | 0.18 |
| Otu31 | *Acinetobacter* | 0.03 | 0.15 | 0.44 | 0.07 | 0.02 | 0.18 | 0.02 |
| Otu10 | *Aeromonas* | 0.00 | 2.30 | 3.93 | 4.63 | 3.44 | 3.99 | 0.00 |
| Otu3 | *Aeromonas* | 0.00 | 0.00 | 1.81 | 1.72 | 20.63 | 8.19 | 0.00 |
| Otu13 | *Bradyrhizobium* | 0.00 | 0.03 | 2.36 | 1.91 | 0.65 | 1.63 | 0.26 |
| Otu6 | *Burkholderia*  *Caballeronia-*  *Paraburkholderia* | 0.00 | 0.00 | 4.23 | 4.58 | 2.00 | 3.58 | 1.91 |
| Otu9 | *Burkholderia*  *Caballeronia-*  *Paraburkholderia* | 0.00 | 0.28 | 11.19 | 0.00 | 0.00 | 3.67 | 0.00 |
| Otu8 | *Caulobacter* | 0.00 | 0.00 | 3.03 | 2.01 | 0.77 | 1.92 | 0.35 |
| Otu65 | *Citrobacter* | 19.50 | 3.45 | 12.46 | 4.81 | 1.75 | 6.27 | 0.00 |
| Otu22 | *Commensalibacter* | 0.00 | 0.76 | 0.00 | 0.00 | 0.00 | 0.00 | 0.00 |
| Otu168 | *Enterobacter* | 0.29 | 0.25 | 0.72 | 0.12 | 0.09 | 0.31 | 0.00 |
| Otu2 | *Enterobacter* | 37.86 | 13.13 | 32.93 | 36.12 | 29.45 | 32.80 | 19.41 |
| Otu52 | *Enterobacter* | 0.00 | 0.00 | 0.15 | 0.07 | 0.77 | 0.34 | 0.00 |
| Otu35 | *Enterococcus* | 0.01 | 0.62 | 0.47 | 0.00 | 0.00 | 0.15 | 0.06 |
| Otu5 | *Enterococcus* | 0.00 | 2.56 | 4.85 | 2.96 | 1.45 | 3.07 | 0.00 |
| Otu124 | *Klebsiella* | 14.80 | 8.20 | 2.94 | 0.33 | 0.00 | 1.08 | 0.00 |
| Otu11 | *Lactobacillus* | 1.57 | 0.54 | 6.02 | 0.00 | 0.00 | 1.98 | 0.00 |
| Otu12 | *Lactococcus* | 2.74 | 0.00 | 0.04 | 3.42 | 0.00 | 1.14 | 0.00 |
| Otu17 | *Mesorhizobium* | 0.00 | 0.00 | 0.95 | 0.78 | 0.36 | 0.69 | 0.04 |
| Otu21 | *Mesorhizobium* | 0.00 | 0.02 | 0.30 | 0.27 | 0.22 | 0.26 | 0.13 |
| Otu26 | *Neisseria* | 0.00 | 0.35 | 1.23 | 0.00 | 0.00 | 0.41 | 0.00 |
| Otu44 | *Neisseria* | 0.00 | 0.00 | 0.52 | 0.03 | 0.03 | 0.19 | 0.00 |
| Otu4 | *Providencia* | 1.38 | 16.56 | 0.27 | 0.26 | 0.00 | 0.18 | 27.91 |
| Otu15 | *Raoultella* | 21.75 | 0.00 | 0.36 | 0.00 | 0.00 | 0.12 | 0.06 |
| Otu136 | *Rheinheimera* | 0.06 | 0.10 | 0.18 | 0.04 | 0.33 | 0.18 | 0.04 |
| Otu16 | *Serratia* | 0.00 | 1.72 | 0.10 | 0.07 | 0.00 | 0.06 | 0.00 |
| Otu14 | *Sphingomonas* | 0.00 | 0.22 | 1.29 | 1.27 | 0.37 | 0.97 | 0.52 |
| Otu20 | *Staphylococcus* | 0.00 | 0.21 | 0.86 | 2.22 | 0.05 | 1.03 | 0.03 |
| Otu7 | *Staphylococcus* | 0.00 | 0.00 | 0.00 | 5.71 | 0.06 | 1.91 | 0.00 |
| Otu24 | *Streptococcus* | 0.00 | 0.48 | 0.77 | 0.10 | 0.10 | 0.32 | 0.00 |
| Otu19 | *Unknown*  *Saccharibacteria* | 0.00 | 1.12 | 0.00 | 0.00 | 0.00 | 0.00 | 0.00 |
| Otu1 | *Wolbachia* | 0.00 | 46.28 | 5.58 | 26.44 | 37.45 | 23.35 | 49.06 |

**C.**

| **OTU ID** | **OTU ID (Genus classification)** | **OTU representation (% of read counts)** | | | | | | | | | |
| --- | --- | --- | --- | --- | --- | --- | --- | --- | --- | --- | --- |
|  |  | **F0**  **T** | **F0**  **PT** | **F1**  **T** | **F1**  **PT** | **F3**  **T** | **F3**  **PT** | **F6**  **T** | **F6**  **PT** | **Lab**  **T** | **Lab**  **PT** |
| Otu18 | *Acinetobacter* | 1,41 | 0,00 | 0,04 | 0,00 | 0,11 | 0,00 | 0,01 | 0,00 | 0,37 | 0,00 |
| Otu31 | *Acinetobacter* | 0,32 | 0,00 | 0,92 | 0,00 | 0,15 | 0,00 | 0,04 | 0,00 | 0,05 | 0,00 |
| Otu10 | *Aeromonas* | 0,00 | 4,36 | 0,00 | 7,55 | 0,00 | 9,08 | 0,15 | 6,64 | 0,00 | 0,00 |
| Otu3 | *Aeromonas* | 0,00 | 0,00 | 3,77 | 0,00 | 3,51 | 0,00 | 0,75 | 39,95 | 0,00 | 0,00 |
| Otu13 | *Bradyrhizobium* | 0,00 | 0,05 | 4,93 | 0,00 | 3,90 | 0,00 | 1,31 | 0,00 | 0,52 | 0,00 |
| Otu6 | *Burkholderia-*  *Caballeronia-*  *Paraburkholderia* | 0,00 | 0,00 | 8,83 | 0,00 | 9,34 | 0,00 | 4,05 | 0,00 | 3,81 | 0,00 |
| Otu9 | *Burkholderia-*  *Caballeronia-*  *Paraburkholderia* | 0,00 | 0,53 | 23,37 | 0,00 | 0,00 | 0,00 | 0,00 | 0,00 | 0,00 | 0,00 |
| Otu8 | *Caulobacter* | 0,00 | 0,00 | 6,32 | 0,00 | 4,11 | 0,00 | 1,57 | 0,00 | 0,70 | 0,00 |
| Otu65 | *Citrobacter* | 0,53 | 6,06 | 2,01 | 22,06 | 0,37 | 9,07 | 3,55 | 0,00 | 0,00 | 0,00 |
| Otu22 | *Commensalibacter* | 0,00 | 1,43 | 0,00 | 0,00 | 0,00 | 0,00 | 0,00 | 0,00 | 0,00 | 0,00 |
| Otu168 | *Enterobacter* | 0,00 | 0,47 | 0,11 | 1,28 | 0,04 | 0,20 | 0,00 | 0,18 | 0,00 | 0,00 |
| Otu2 | *Enterobacter* | 0,00 | 24,84 | 21,89 | 43,08 | 2,06 | 68,88 | 25,95 | 32,86 | 0,00 | 39,02 |
| Otu52 | *Enterobacter* | 0,00 | 0,00 | 0,31 | 0,00 | 0,14 | 0,00 | 0,00 | 1,53 | 0,00 | 0,00 |
| Otu35 | *Enterococcus* | 0,90 | 0,38 | 0,02 | 0,89 | 0,00 | 0,00 | 0,00 | 0,00 | 0,00 | 0,12 |
| Otu5 | *Enterococcus* | 0,00 | 4,85 | 0,49 | 8,86 | 0,24 | 5,58 | 0,00 | 2,86 | 0,00 | 0,00 |
| Otu124 | *Klebsiella* | 0,00 | 15,50 | 1,04 | 4,69 | 0,26 | 0,41 | 0,00 | 0,00 | 0,00 | 0,00 |
| Otu11 | *Lactobacillus* | 0,00 | 1,02 | 0,00 | 11,56 | 0,00 | 0,00 | 0,00 | 0,00 | 0,00 | 0,00 |
| Otu12 | *Lactococcus* | 0,00 | 0,00 | 0,08 | 0,00 | 0,00 | 6,71 | 0,00 | 0,00 | 0,00 | 0,00 |
| Otu17 | *Mesorhizobium* | 0,00 | 0,00 | 1,99 | 0,00 | 1,59 | 0,00 | 0,72 | 0,00 | 0,08 | 0,00 |
| Otu21 | *Mesorhizobium* | 0,00 | 0,03 | 0,62 | 0,00 | 0,54 | 0,00 | 0,46 | 0,00 | 0,25 | 0,00 |
| Otu26 | *Neisseria* | 0,75 | 0,00 | 2,58 | 0,00 | 0,00 | 0,00 | 0,00 | 0,00 | 0,00 | 0,00 |
| Otu44 | *Neisseria* | 0,01 | 0,00 | 1,08 | 0,00 | 0,07 | 0,00 | 0,06 | 0,00 | 0,00 | 0,00 |
| Otu4 | *Providencia* | 0,55 | 30,84 | 0,57 | 0,00 | 0,53 | 0,00 | 0,00 | 0,00 | 0,00 | 56,09 |
| Otu15 | *Raoultella* | 0,00 | 0,00 | 0,74 | 0,00 | 0,00 | 0,00 | 0,00 | 0,00 | 0,00 | 0,13 |
| Otu136 | *Rheinheimera* | 0,11 | 0,08 | 0,34 | 0,03 | 0,00 | 0,08 | 0,36 | 0,29 | 0,00 | 0,08 |
| Otu16 | *Serratia* | 0,50 | 2,80 | 0,21 | 0,00 | 0,14 | 0,00 | 0,00 | 0,00 | 0,00 | 0,00 |
| Otu14 | *Sphingomonas* | 0,46 | 0,00 | 2,70 | 0,00 | 2,58 | 0,00 | 0,75 | 0,00 | 1,04 | 0,00 |
| Otu20 | *Staphylococcus* | 0,40 | 0,04 | 1,79 | 0,00 | 4,53 | 0,00 | 0,10 | 0,00 | 0,07 | 0,00 |
| Otu7 | *Staphylococcus* | 0,00 | 0,00 | 0,00 | 0,00 | 11,66 | 0,00 | 0,12 | 0,00 | 0,00 | 0,00 |
| Otu24 | *Streptococcus* | 1,01 | 0,00 | 1,61 | 0,00 | 0,20 | 0,00 | 0,21 | 0,00 | 0,00 | 0,00 |
| Otu19 | *Unknown Saccharibacteria* | 2,38 | 0,00 | 0,00 | 0,00 | 0,00 | 0,00 | 0,00 | 0,00 | 0,00 | 0,00 |
| Otu1 | *Wolbachia* | 90,67 | 6,70 | 11,65 | 0,00 | 53,93 | 0,00 | 59,82 | 15,70 | 93,12 | 4,57 |

**D.**

| **OTU**  **ID** | **OTU ID (Genus classification)** | **OTU representation (% of samples)** | | | | | | | | | |
| --- | --- | --- | --- | --- | --- | --- | --- | --- | --- | --- | --- |
|  |  | **F0**  **T** | **F0**  **PT** | **F1**  **T** | **F1**  **PT** | **F3**  **T** | **F3**  **PT** | **F6**  **T** | **F6**  **PT** | **Lab**  **T** | **Lab**  **PT** |
| Otu18 | *Acinetobacter* | 100,00 | 0,00 | 16,67 | 0,00 | 33,33 | 0,00 | 16,67 | 0,00 | 50,00 | 0,00 |
| Otu31 | *Acinetobacter* | 100,00 | 0,00 | 50,00 | 0,00 | 83,33 | 0,00 | 33,33 | 0,00 | 83,33 | 0,00 |
| Otu10 | *Aeromonas* | 0,00 | 33,33 | 0,00 | 16,67 | 0,00 | 50,00 | 16,67 | 66,67 | 0,00 | 0,00 |
| Otu3 | *Aeromonas* | 0,00 | 0,00 | 50,00 | 0,00 | 66,67 | 0,00 | 33,33 | 83,33 | 0,00 | 0,00 |
| Otu13 | *Bradyrhizobium* | 0,00 | 16,67 | 100,00 | 0,00 | 100,00 | 0,00 | 100,00 | 0,00 | 83,33 | 0,00 |
| Otu6 | *Burkholderia-*  *Caballeronia-*  *Paraburkholderia* | 0,00 | 0,00 | 83,33 | 0,00 | 100,00 | 0,00 | 100,00 | 0,00 | 100,00 | 0,00 |
| Otu9 | *Burkholderia-*  *Caballeronia-*  *Paraburkholderia* | 0,00 | 33,33 | 66,67 | 0,00 | 0,00 | 0,00 | 0,00 | 0,00 | 0,00 | 0,00 |
| Otu8 | *Caulobacter* | 0,00 | 0,00 | 100,00 | 0,00 | 100,00 | 0,00 | 100,00 | 0,00 | 100,00 | 0,00 |
| Otu65 | *Citrobacter* | 16,67 | 100,00 | 33,33 | 100,00 | 16,67 | 83,33 | 16,67 | 0,00 | 0,00 | 0,00 |
| Otu22 | *Commensalibacter* | 0,00 | 50,00 | 0,00 | 0,00 | 0,00 | 0,00 | 0,00 | 0,00 | 0,00 | 0,00 |
| Otu168 | *Enterobacter* | 0,00 | 100,00 | 33,33 | 100,00 | 16,67 | 33,33 | 0,00 | 100,00 | 0,00 | 0,00 |
| Otu2 | *Enterobacter* | 0,00 | 100,00 | 50,00 | 100,00 | 16,67 | 100,00 | 50,00 | 100,00 | 0,00 | 83,33 |
| Otu52 | *Enterobacter* | 0,00 | 0,00 | 33,33 | 0,00 | 16,67 | 0,00 | 0,00 | 83,33 | 0,00 | 0,00 |
| Otu35 | *Enterococcus* | 100,00 | 33,33 | 16,67 | 83,33 | 0,00 | 0,00 | 0,00 | 0,00 | 0,00 | 66,67 |
| Otu5 | *Enterococcus* | 0,00 | 83,33 | 16,67 | 100,00 | 16,67 | 66,67 | 0,00 | 100,00 | 0,00 | 0,00 |
| Otu124 | *Klebsiella* | 0,00 | 66,67 | 33,33 | 100,00 | 16,67 | 33,33 | 0,00 | 0,00 | 0,00 | 0,00 |
| Otu11 | *Lactobacillus* | 0,00 | 33,33 | 0,00 | 100,00 | 0,00 | 0,00 | 0,00 | 0,00 | 0,00 | 0,00 |
| Otu12 | *Lactococcus* | 0,00 | 0,00 | 16,67 | 0,00 | 0,00 | 83,33 | 0,00 | 0,00 | 0,00 | 0,00 |
| Otu17 | *Mesorhizobium* | 0,00 | 0,00 | 66,67 | 0,00 | 100,00 | 0,00 | 100,00 | 0,00 | 50,00 | 0,00 |
| Otu21 | *Mesorhizobium* | 0,00 | 33,33 | 83,33 | 0,00 | 100,00 | 0,00 | 100,00 | 0,00 | 100,00 | 0,00 |
| Otu26 | *Neisseria* | 66,67 | 0,00 | 16,67 | 0,00 | 0,00 | 0,00 | 0,00 | 0,00 | 0,00 | 0,00 |
| Otu44 | *Neisseria* | 16,67 | 0,00 | 16,67 | 0,00 | 16,67 | 0,00 | 33,33 | 0,00 | 0,00 | 0,00 |
| Otu4 | *Providencia* | 16,67 | 66,67 | 16,67 | 0,00 | 16,67 | 0,00 | 0,00 | 0,00 | 0,00 | 100,00 |
| Otu15 | *Raoultella* | 0,00 | 0,00 | 16,67 | 0,00 | 0,00 | 0,00 | 0,00 | 0,00 | 0,00 | 16,67 |
| Otu136 | *Rheinheimera* | 33,33 | 50,00 | 33,33 | 16,67 | 0,00 | 83,33 | 50,00 | 100,00 | 0,00 | 66,67 |
| Otu16 | *Serratia* | 100,00 | 33,33 | 50,00 | 0,00 | 50,00 | 0,00 | 0,00 | 0,00 | 0,00 | 0,00 |
| Otu14 | *Sphingomonas* | 100,00 | 0,00 | 66,67 | 0,00 | 100,00 | 0,00 | 66,67 | 0,00 | 100,00 | 0,00 |
| Otu20 | *Staphylococcus* | 66,67 | 16,67 | 66,67 | 0,00 | 100,00 | 0,00 | 33,33 | 0,00 | 16,67 | 0,00 |
| Otu7 | *Staphylococcus* | 0,00 | 0,00 | 0,00 | 0,00 | 50,00 | 0,00 | 16,67 | 0,00 | 0,00 | 0,00 |
| Otu24 | *Streptococcus* | 83,33 | 0,00 | 50,00 | 0,00 | 83,33 | 0,00 | 66,67 | 0,00 | 0,00 | 0,00 |
| Otu19 | *Unknown Saccharibacteria* | 100,00 | 0,00 | 0,00 | 0,00 | 0,00 | 0,00 | 0,00 | 0,00 | 0,00 | 0,00 |
| Otu1 | *Wolbachia* | 100,00 | 50,00 | 50,00 | 0,00 | 100,00 | 0,00 | 100,00 | 100,00 | 100,00 | 33,33 |
