# Supplementary material for: Gut Bacteriome Analysis of Anastrepha fraterculus sp. 1 During the Early Steps of Laboratory Colonization
Source: Front Microbiol. 2020 Oct 20;11:570960. doi: 10.3389/fmicb.2020.570960 (PMC7606190; doi:10.3389/fmicb.2020.570960)
Supplement: Supplementary Table 3 — Bacterial diversity and richness (Shannon and Chao indices). [file Table_3.DOC]

| **Generation_feeding status_sex** | **Number of Reads** | **Chao Index** | **Shannon Effective Index** |
| --- | --- | --- | --- |
| F0_T_F | 44935 | 10 | 0.32 |
| F0_T_F | 43796 | 10 | 0.69 |
| F0_T_F | 45394 | 8 | 0.29 |
| F0_T_M | 43704 | 12 | 0.58 |
| F0_T_M | 40260 | 10 | 0.64 |
| F0_T_M | 44453 | 10 | 0.35 |
| F0_PT_M | 49452 | 8 | 1.24 |
| F0_PT_M | 48725 | 6 | 1.02 |
| F0_PT_M | 48652 | 11 | 1.87 |
| F0_PT_F | 48550 | 9 | 0.72 |
| F0_PT_F | 49736 | 10 | 1.62 |
| F0_PT_F | 49270 | 10 | 1.63 |
| F1_T_M | 44390 | 14 | 2.02 |
| F1_T_M | 45465 | 13 | 1.27 |
| F1_T_M | 47607 | 18 | 2.04 |
| F1_T_F | 40544 | 11 | 2.18 |
| F1_T_F | 48955 | 10 | 0.70 |
| F1_T_F | 44001 | 8 | 1.16 |
| F1_PT_M | 49131 | 7 | 1.40 |
| F1_PT_M | 49535 | 8 | 1.34 |
| F1_PT_M | 49313 | 7 | 1.46 |
| F1_PT_F | 49172 | 7 | 1.46 |
| F1_PT_F | 49020 | 7 | 1.31 |
| F1_PT_F | 48554 | 7 | 1.14 |
| F3_T_M | 44999 | 18 | 2.04 |
| F3_T_M | 46474 | 14 | 1.89 |
| F3_T_M | 46460 | 11 | 1.32 |
| F3_T_F | 46537 | 12 | 1.12 |
| F3_T_F | 47796 | 12 | 1.34 |
| F3_T_F | 47521 | 11 | 0.85 |
| F3_PT_M | 48337 | 6 | 1.24 |
| F3_PT_M | 48743 | 8 | 1.15 |
| F3_PT_M | 48410 | 3 | 0.29 |
| F3_PT_F | 48372 | 4 | 0.19 |
| F3_PT_F | 49093 | 6 | 1.10 |
| F3_PT_F | 48008 | 5 | 0.87 |
| F6_T_M | 47565 | 11 | 1.07 |
| F6_T_M | 45149 | 14 | 0.91 |
| F6_T_M | 49353 | 9 | 0.82 |
| F6_T_F | 49452 | 10 | 1.18 |
| F6_T_F | 48835 | 7 | 0.32 |
| F6_T_F | 49387 | 11 | 0.97 |
| F6_PT_M | 49143 | 8 | 1.26 |
| F6_PT_M | 49492 | 7 | 0.99 |
| F6_PT_M | 49853 | 8 | 1.13 |
| F6_PT_F | 49902 | 7 | 1.11 |
| F6_PT_F | 49776 | 6 | 1.07 |
| F6_PT_F | 49876 | 8 | 1.27 |
| Lab_T_M | 47334 | 10 | 0.50 |
| Lab_T_M | 48317 | 8 | 0.32 |
| Lab_T_M | 46957 | 8 | 0.39 |
| Lab_T_F | 48501 | 9 | 0.37 |
| Lab_T_F | 48360 | 7 | 0.25 |
| Lab_T_F | 49429 | 5 | 0.21 |
| Lab_PT_M | 48890 | 5 | 1.02 |
| Lab_PT_M | 44476 | 1 | 0.00 |
| Lab_PT_M | 47334 | 3 | 0.69 |
| Lab_PT_F | 48170 | 3 | 0.65 |
| Lab_PT_F | 49604 | 6 | 0.99 |
| Lab_PT_F | 47566 | 4 | 0.71 |
| WU_unk_M | 48457 | 7 | 1.30 |
| WU_unk_M | 49320 | 6 | 0.93 |
| WU_unk_M | 49148 | 6 | 0.82 |
| WU_unk_F | 47737 | 8 | 1.05 |
| WU_unk_F | 48837 | 9 | 1.52 |
| WU_unk_F | 48081 | 7 | 1.06 |
