# Supplementary material for: Gut Bacteriome Analysis of Anastrepha fraterculus sp. 1 During the Early Steps of Laboratory Colonization
Source: Front Microbiol. 2020 Oct 20;11:570960. doi: 10.3389/fmicb.2020.570960 (PMC7606190; doi:10.3389/fmicb.2020.570960)
Supplement: Supplementary Table 4 — Relative abundance of Wolbachia (OTU 1) pair-wise comparisons (teneral [T] vs post-teneral [PT]) using Wilcoxon Rank Sum Test. Group compared: generation F0–F6 and laboratory flies (Lab). Mean values of each compared group were considered to the statistical analysis. [file Table_4.DOC]

| **OTU ID** | **Group1 (PT)** | **Group2 (T)** | **p value** | **Corrected p value** |
| --- | --- | --- | --- | --- |
| Otu1 | F0_PT | F0_T | 0,0048 | 0,0078 |
| Otu1 | F1_PT | F1_T | 0,074 | 0,093 |
| Otu1 | F3_PT | F3_T | 0,0028 | 0,0077 |
| Otu1 | F6_PT | F6_T | 0,0022 | 0,0077 |
| Otu1 | Lab_PT | Lab_T | 0,0043 | 0,0078 |
